# Cotton microbiome profiling and Cotton Leaf Curl Disease (CLCuD) suppression through microbial consortia associated with *Gossypium arboreum*

**DOI:** 10.1038/s41522-023-00470-9

**Published:** 2023-12-14

**Authors:** Rhea Aqueel, Ayesha Badar, Nazish Roy, Qandeel Mushtaq, Aimen Fatima Ali, Aftab Bashir, Umer Zeeshan Ijaz, Kauser Abdulla Malik

**Affiliations:** 1 Kauser Abdulla Malik School of Life Sciences, Forman Christian College (A Chartered University), Ferozepur Road, Lahore, 54600 Pakistan; 2 Water & Environment Research Group, University of Glasgow, Mazumdar-Shaw Advanced Research Centre, Glasgow, G11 6EW UK; 3 National University of Ireland, Galway, University Road, Galway, H91 TK33 Ireland; 4 Department of Molecular and Clinical Cancer Medicine, University of Liverpool, Liverpool, L69 7BE UK; 5 Pakistan Academy of Sciences, Islamabad, Pakistan

**Keywords:** Applied microbiology, Microbiome

## Abstract

The failure of breeding strategies has caused scientists to shift to other means where the new approach involves exploring the microbiome to modulate plant defense mechanisms against Cotton Leaf Curl Disease (CLCuD). The cotton microbiome of CLCuD-resistant varieties may harbor a multitude of bacterial genera that significantly contribute to disease resistance and provide information on metabolic pathways that differ between the susceptible and resistant varieties. The current study explores the microbiome of CLCuD-susceptible *Gossypium hirsutum* and CLCuD-resistant *Gossypium arboreum* using 16 S rRNA gene amplification for the leaf endophyte, leaf epiphyte, rhizosphere, and root endophyte of the two cotton species. This revealed that *Pseudomonas* inhabited the rhizosphere while *Bacillus* was predominantly found in the phyllosphere of CLCuV-resistant *G. arboreum*. Using salicylic acid-producing *Serratia spp*. and *Fictibacillus spp*. isolated from CLCuD-resistant *G. arboreum,* and guided by our analyses, we have successfully suppressed CLCuD in the susceptible *G. hirsutum* through pot assays. The applied strains exhibited less than 10% CLCuD incidence as compared to control group where it was 40% at 40 days post viral inoculation. Through detailed analytics, we have successfully demonstrated that the applied microbes serve as a biocontrol agent to suppress viral disease in Cotton.

## Introduction

Cotton Leaf Curl Disease (CLCuD), transmitted by the whitefly *Bemisia tabaci*, has devastated Pakistan’s cotton crop for the past three decades^1,2^. Overall insect pests account for 37% of cotton yield losses, whereas *Bemisia tabaci* is responsible for 50% of the total loss in cotton production due to insects^3,4^. A few CLCuD-resistant lines of *Gossypium arboreum* have been developed through conventional breeding approaches, but the worldwide extensively cultivated *Gossypium hirsutum* remains highly susceptible to CLCuD^5,6^.

Plant-associated microbial communities reveal much about the correlation between microbiome composition and functional profiles^7^. The potential of the rhizosphere microbiome in suppressing fungal and bacterial diseases has been tapped^8,9^, but the plant microbiome’s role in suppressing viral diseases is still unexplored^10^. The plant microbiome functions as one of the key determinants of plant health and productivity by providing defense against environmental stress and pathogens. Biotic or abiotic factors affecting plant-microbe interactions contribute to distinct microbial communities spread in the three plant regions, namely rhizosphere, phyllosphere, and endosphere^11,12^. The microbiome of cotton plants is highly affected by their genotype and developmental stages^13^. Each cultivar’s production of genotype-specific root exudates recruits specific types of microbial communities. Similarly, at various developmental stages, different plant hormones recruit specific microbes. Recent studies suggest that variation in plant genotypes, even at a single gene locus, can significantly impact rhizosphere microbiomes^14,15^.

Plant phyllosphere microbiotas play a crucial role in activating plants’ antioxidant status by producing pathogenesis-related (PR) proteins and demonstrate their capability as a promising approach for disease control management and induction of systemic resistance^16,17^. At the phylum level, the phyllosphere bacterial communities typically include Actinobacteria, Bacteroidetes, Firmicutes, and Proteobacteria, dominated by Alpha proteobacteria and Gamma proteobacteria^18,19^. Plant secondary metabolites such as glucosinolates influence bacterial colonization in the phyllosphere and exhibit plant pathogen-inhibiting potential^20^. The phytohormone Salicylic Acid (SA) has also been studied as a protective regime against CLCuD in cotton plants where less incidence of disease and increased PR protein expression were reported^21^. The SA signaling pathway is critical to immunity against biotrophic pathogens^22^. The endosphere-associated microbes aid in plant growth and development by modulating pathways involved in plant defense and metabolism. These metabolic interactions are crucial in enhancing nutrient uptake and providing plant tolerance against biotic and abiotic stresses^23,24^.

Using the sensitive approach of next-generation sequencing-based metagenomics, we conducted complex microbial community analyses of the two cotton species, *Gossypium hirsutum*, and *Gossypium arboreum*, under CLCuD attack. We aim to highlight the role of the microbiome in suppressing plant viral disease, which has yet to be elucidated. The microbiome’s diversity reflects the correlation between microbial composition and metabolic pathways playing key roles in the cotton plant under Cotton Leaf Curl Virus (CLCuV) infection.

## Results

### Comparative microbiome analysis shows significant variability in microbial community structure based on cotton species, varieties, and compartments

**Fig. 1:**
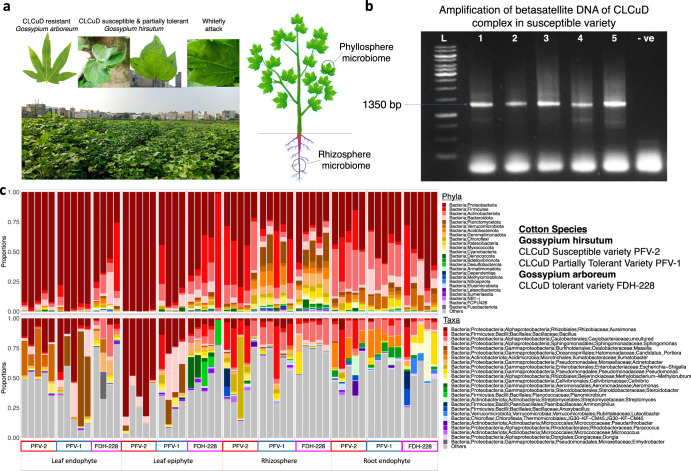
Microbiome profiles of CLCuD susceptible, partially tolerant and resistant cotton varieties. **a** CLCuD-infected cotton plants. **b** Gel Image showing beta-satellite DNA amplification for CLCuV confirmation in cotton plants. **c** Taxonomy Summary at Phylum and Genus Level for *Gossypium hirsutum* and *Gossypium arboreum* infected with CLCuV.

Through Illumina MiSeq sequencing, 38,120 ASVs were identified among 59 samples for the phyllospheric and rhizospheric bacterial community of CLCuV-infected susceptible and resistant cotton plants. The composition of bacterial phyla and the predominant genera of the different compartments of CLCuD susceptible and resistant cotton species can be seen in Fig. 1. Analysis of four compartments (LE, LN, RZ, RN) of *Gossypium hirsutum* and *Gossypium arboreum* at phyla level revealed the abundance of top 5 abundant phyla as Proteobacteria, Firmicutes, Actinobacteria, Bacteroidota, Planctomycetota (Fig. 1c). The phyla dominating *the G. hirsutum* phyllosphere were Proteobacteria, Firmicutes, Actinobacteria, and Bacteroidota. *G. arboreum* phyllosphere had Proteobacteria and Firmicutes as dominant phyla, but it also had other phyla like Gemmatimonadota and Planctomycetota, which were absent in the *G. hirsutum* phyllosphere. Planctomycetota was among the dominant phyla in the RZ of both *G. hirsutum* and *G. arboreum*, others being Myxococcota, Verrucomicrobiota, and Deinococcota. The RN of *G. hirsutum* was dominated by Proteobacteria and Actinobacteriota, whereas in *G. arboreum*, Actinobacteriota was not that dominant. The most abundant bacterial genera in CLCuD susceptible and resistant cotton species are depicted in Fig. 1c. *Aureimonas* and *Massilia* were the predominant genera in the phyllosphere of *G. hirsutum*. However, *Candidatus portiera* was only observed in the LN region. The RZ of *G. hirsutum* included *Caulobacteraceae, Bacillus, and Escherichia-Shigella*, whereas, *Steroidobacter* and *Streptomyces* were found in the RN only. *Caulobacteraceae* and *Acinetobacter* were present in all four compartments of *G. arboreum*. *Planomicrobium* was in the LE region only. *Pseudomonas* was dominant in the RZ. *Ammoniphilus, Anoxybacillus*, and *Luteolibacter* were abundant in the RN region of *G. arboreum only*.

**Fig. 2:**
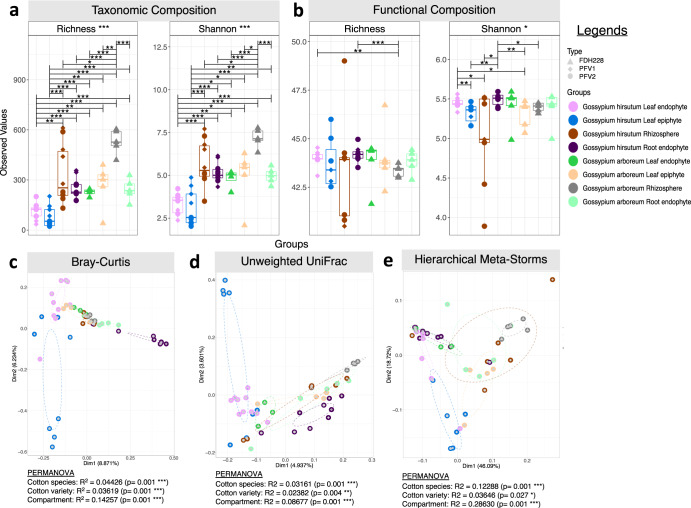
Alpha and Beta diversity results for the cotton microbiome under CLCuD attack. Alpha diversity indices for (**a**) Microbial composition; and (**b**) MetaCyc pathways returned from PICRUSt2 procedure. Lines for panels connect two sample groups at statistically significant levels (ANOVA) indicated by asterisks as *p < 0.05, **p < 0.01, or ***p < 0.001. Beta diversity represented through principal coordinate analysis (PCoA) plots calculated using (**c**) Bray–Curtis, (**d**) unweighted UniFrac, and (**e**) hierarchical meta-storms distances. The ellipses represent the 95% confidence interval of the standard error of the ordination points of a given grouping. Beneath the figures, PERMANOVA R^2^ explains significant variability in microbial community structure based on Cotton species, varieties, and compartments.

Shannon and Richness, alpha diversity measures, were used for taxonomic composition. Richness is the number of ASVs/species when samples are rarefied (Fig. 2a). The highest value (528.84 ± 74.13) in taxonomy was observed in the RZ of the CLCuD-resistant FDH-228. The LE of CLCuD susceptible PFV-2 exhibited the lowest richness (34.50 ± 13.11). However, the partially tolerant variety PFV-1 within *Gossypium hirsutum* species was higher than PFV-2 itself. The observed ASVs, Shannon, and richness of the bacterial communities revealed significant differences between phyllospheric and rhizospheric bacteria of the three cotton varieties. Richness in metabolic functions (Fig. 2b) was higher in *Gossypium hirsutum* than in *Gossypium arboreum*. Significant results were obtained for *G. hirsutum* LN and *G. hirsutum* RN in comparison with *G. arboreum* RZ, respectively. Based on taxonomic abundance, the most balanced microbial communities belonged to the RZ of both susceptible and resistant cotton species (Fig. 2a). In terms of functions, all compartments depicted balance, with *G. arboreum* LN and *G. hirsutum* RN being the highest (Fig. 2b).

Sample dissimilarity was depicted using beta diversity measures, including Bray–Curtis distance for compositional changes (Fig. 2c), Unweighted UniFrac distance for phylogenetic changes (Fig. 2d), and Hierarchical Meta-Storms for changes in metabolic functions (Fig. 2e). It can be observed that among *Gossypium hirsutum* LE, there are two distinct groups clustered far apart of the susceptible (PFV-2) and partially tolerant (PFV-1) varieties in terms of taxa and phylogeny. Permutational multivariate analysis of variance (PERMANOVA) revealed significant variability in compartments, with 28% variability in function, 14% in composition, and 8% in phylogeny. Among cotton species, the highest source of variability was in terms of function (12%) as compared to composition (4%) and phylogeny (3%). For the three cotton varieties, 3% variability was observed in composition and functions, respectively, while 2% variability was observed in phylogeny. Unweighted Unifrac and Hierarchical Meta-storms, species, and compartments significantly contributed to the beta diversity among samples.

### The core microbiome of the CLCuD susceptible and resistant cotton species identified at the genus level

**Fig. 3:**
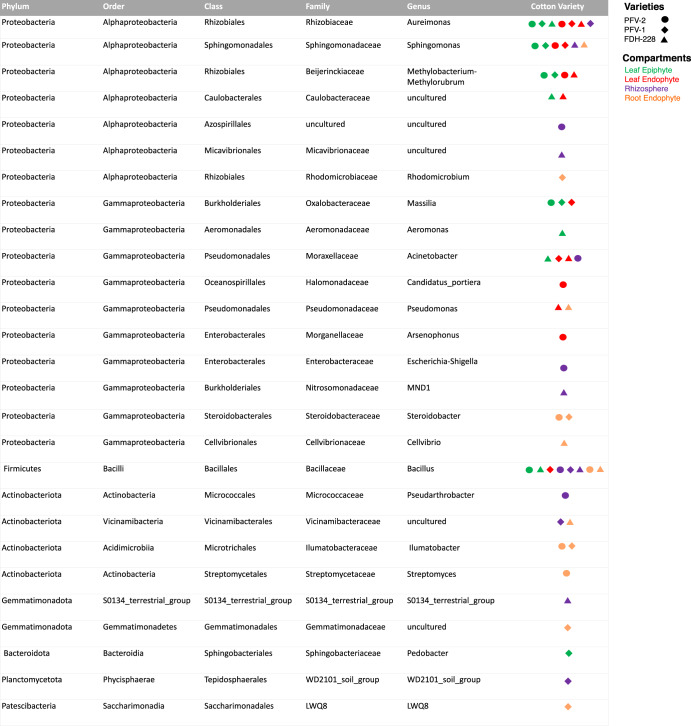
Top five most abundant core microbiome found in each plant Compartment of CLCuD susceptible, partially tolerant and resistant cotton varieties. These are based on Supplementary Figs. 2–4 and represent the core microbiome at the right side of the heatmaps.

A core microbiome represents a set of microbes that persists in a host’s internal or surrounding environment despite location or developmental stage changes. In the CLCuD susceptible variety PFV-2 (Supplementary Fig. 2), the bacterial core of LE contained 17 ASVs with *Aureimonas, Sphingomonas, Methylorubrum, Bacillus*, and *Massilia* as the most prevalent genera. *Candidatus portiera, Aureimonas, Arsenophonus, Sphingomonas*, and *Methylorubrum* dominated the LN region out of 31 ASVs. The RZ contained 26 ASVs, with *Bacillus, Pseudarthrobacter, Azospirillales, Shigella*, and *Acinetobacter* highlighted as the most prevalent. Of 58 ASVs, *Alphaproteobacteria, Bacillus, Ilumatobacter, Steroidobacter*, and *Streptomyces* were predominantly present in the RN of PFV-2. The LE, LN, and RZ of CLCuD-resistant FDH-228 have a high abundance core microbiome compared to PFV-1 and PFV-2. Overall, the bacterial core of FDH-228 (Supplementary Fig. 4) contained 96 ASVs (RN) with *Vicinamibacterales, Pseudomonas, Sphingomonas, Cellvibrio*, and *Bacillus* as the most predominant genera. The LN compartment (44 ASVs) had *Acinetobacter, Caulobacteraceae, Aureimonas, Methylobacterium-Methylorubrum*, and *Pseudomonas* in prevalence. Of an abundant 224 ASVs in the RZ, the genera *Micavibrionaceae, S0134_terrestrial group, Sphingomonas, MND1*, and *Bacillus* were the most dominant. In the resistant species, *Aureimonas, Caulobacteraceae, Aeromonas, Bacillus*, and *Acinetobacter* were the top abundant bacterial genera among 145 ASVs (LE). Only the RN compartment had a higher abundance core microbiome in partially tolerant PFV-1 (166 ASVs) as compared to PFV-2 (58 ASVs) and FDH-228 (96 ASVs). The top five most abundant core microbiome existing in each of the four plant compartments of the three selected varieties (PFV-2, PFV1, and FDH-228) are depicted in Fig. 3.

### Distinct subsets of bacterial species in CLCuV-infected cotton plant rhizosphere and phyllosphere compartments

The association between the selected cotton plant varieties and compartments with the minimal subset of microbes was studied using the CODA LASSO variable selection approach. Positive or negative associated microbes were identified with the covariate of interest. The bacterial ASV discriminant in the RZ of PFV-1 and FDH-228 are shown in Supplementary Fig. 5, where *Methylophaga* and *Candidatus adlerbacteria* increased in FDH-228. In the LE compartment (Supplementary Fig. 6), *Aeromonas* ASVs were positively associated with the CLCuD-resistant FDH-228, while they were negatively associated with the partially tolerant PFV-1. On the contrary, *Thermus* was positively associated with the partially tolerant variety PFV-1 and showed a negative association with the resistant FDH-228 in the LE region. In the RZ compartment (Supplementary Fig. 8), *Ahniella* and *Nocardia* were positively associated with the CLCuD susceptible variety PFV-2 while showing a negative association with the resistant FDH-228. *Gemmatimonas* was positively associated with FDH-228 in the RZ and negatively associated with the susceptible variety. The significant positive and negative associations of bacterial ASVs associated with different plant compartments among the three cotton varieties have been shown in Supplementary Figs. 5–11. The top 25 genera having significant correlations have been listed in Supplementary Table 3.

### Metabolic pathways abundance in CLCuV-infected cotton plant rhizosphere and phyllosphere

To elucidate the functional disparity between CLCuD susceptible and resistant cotton species, a total of 197 MetaCyc pathways were predicted, including cellular processes, amino acid metabolism, carbohydrate metabolism, and resistance mechanisms, using the PICRUSt2 algorithm. The MetaCyc pathways of the archaea, such as those involved in phosphopantothenate, flavin, and chorismate biosynthesis, are positively associated with the *Gossypium arboreum* RN microbiome as compared to the partially tolerant PFV-1 (Supplementary Fig. 12). Pathways such as formaldehyde assimilation by methanogenic bacteria and phosphopanthothenate biosynthesis by archaea were positively associated with FDH-228 and negatively associated with PFV-2 in the RZ microbiome (Supplementary Fig. 13). The super pathway of salicylate degradation depicts a positive association with the RZ microbiome of susceptible PFV-2, whereas a negative association with resistant FDH-228. Ectoine, Palmitate, and Sucrose biosynthesis pathways were positively associated with the LN microbiome of susceptible PFV-2 as compared to resistant FDH-228 (Supplementary Fig. 14). All detailed pathways showing differential abundance are shown in Supplementary Figs. 12–17.

### Key genera associated with sources of variability

To find the positive or negative association between microbial communities and the sources of variation, we have fitted a regression model, *the Generalised Linear Latent Variable Model* (GLLVM), which also gives us correlations between microbial species after accounting for the confounders (Cotton Species, Cotton Variety, CLCuV Susceptibility, and Compartment) as given in Supplementary Fig. 18. Most of the microbes were positively associated with the RZ and RN plant compartments with reference to LN. The below-ground interactions and possibly root exudate chemistry are major contributing factors to these positive associations with the environmental covariates understudy. Focusing on the SA-producing bacteria, we found all of them: *Sphingomonas, Bacillus, Pseudomonas, Nocardioides*, and *Paenibacillus* to be positively associated with CLCuV resistance. Only *Sphingomonas* was positively associated with the LE in *Gossypium hirsutum*. *Bacillus, Pseudomonas*, and *Nocardioides* were positively associated with the RZ and the partially tolerant variety PFV-1.

### Impact of Intra- and Interspecific variation on plant-microbial associations

**Fig. 4:**
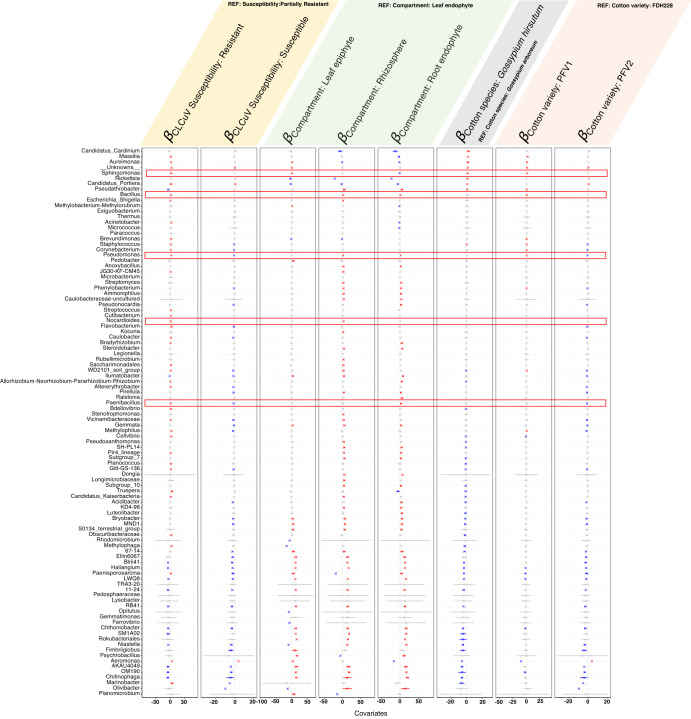
*β-*coefficients returned from GLLVM procedure for covariates considered in this comparative microbiome analysis between *Gossypium arboreum* and *Gossypium hirsutum* under CLCuD attack. Those coefficients which are positively associated with the microbial abundance of a particular species are represented in red color whilst those that are negatively associated are represented with blue color, respectively. Those microbes which were statistically insignificant, i.e., where the coefficients crossed 0 boundary, are grayed out. the Since the collation of ASVs were done at Genus level, all those ASVs that cannot be categorized based on taxonomy are collated under “__Unknowns__” category. The highlighted genera are salicylic acid producing bacteria.

Apart from the obvious interspecific microbial diversity differences, there is no denying the fact that within species the microbiome varies under CLCuD infestation. Among sources of variation as visualized using the GLLVM model (Fig. 4), *Sphingomonas, Bacillus*, and *Pseudomonas* show a positive association with the partially tolerant *G.hirsutum* variety PFV-1 whereas they are negatively associated or statistically insignificant in the highly susceptible *G. hirsutum* variety PFV-2. Comparing the microbiome profiles at the genus level in Fig. 1c. *Massilia* can be observed in the phyllosphere of PFV-1 whereas it is absent in PFV-2. The rhizosphere of PFV-2 has a high abundance of *Escherichia-Shigella*, whereas the rhizosphere of PFV-1 and FDH-228 is somewhat similar. Supplementary Figure 2–4 depict how the core microbiome varies with disease susceptibility of each variety. Although the CLCuD-resistant variety has a dynamic core microbiome and possesses functional relationships with the host plant that contribute to disease resistance, but it is interesting to note that the partially tolerant PFV-1 harbors a multitude of bacterial genera that are absent in the susceptible PFV-2. These intraspecific differences in the microbial composition therefore play a crucial role in their response mechanisms towards CLCuD.

### The microbiome of Gossypium arboreum mediates viral disease suppression in Gossypium hirsutum

**Fig. 5:**
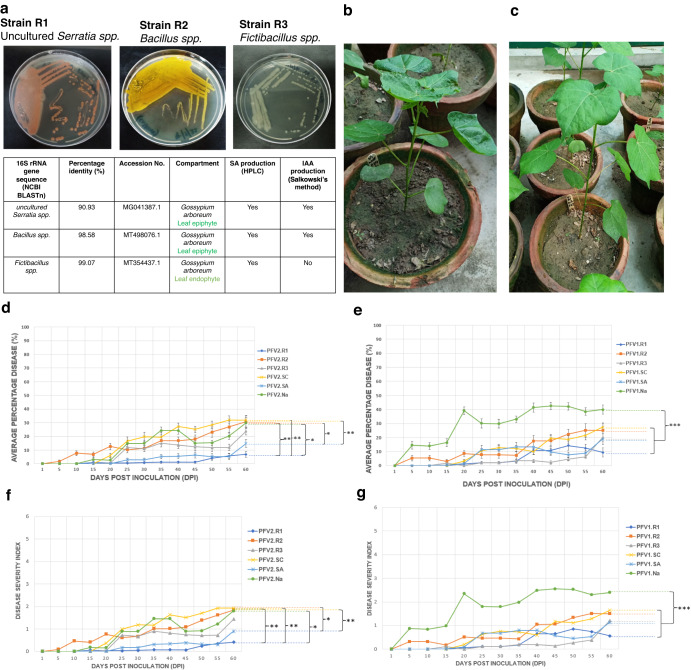
Microbiome mediated CLCuD suppression under SA producing strains Application. **a** Salicylic Acid producing strains isolated from CLCuD resistant variety FDH-228. **b** *Gossypium hirsutum* PFV-1 control plant with no application (nA) exhibiting CLCuD symptoms at 20 DPI. **c** *Gossypium hirsutum* PFV-1 plant with application of strain R1 (*uncultured Serratia spp*.) resisting disease at 20 DPI. **d** CLCuD disease progression in Susceptible variety (PFV-2) under different applications: PFV2.R1 (*uncultured Serratia spp*.), PFV2.R2 (*Bacillus spp*.), PFV2.R3 (*Fictibacillus spp.)*, PFV2.SC (Synthetic consortium of R1, R2, R3), PFV2.SA (Salicylic acid 400 mg/L positive control), PFV2.NA (No application- control) Statistically significant levels (Tukey’s HSD post hoc test) are indicated by asterisks as *p < 0.05, **p < 0.01, or ***p < 0.001. **e** CLCuD disease progression in Partially tolerant variety (PFV-1) under different applications: PFV1.R1 (*uncultured Serratia spp*.), PFV1.R2 (*Bacillus spp*.), PFV1.R3 (*Fictibacillus spp*.), PFV1.SC (Synthetic consortium of R1, R2, R3), PFV1.SA (Salicylic acid 400 mg/L positive control), PFV1.NA (No application- control). **f**, **g** are similar to **d** and **e** except that we have represented the results in disease severity index. Statistically significant levels (Tukey’s HSD post hoc test) are indicated by asterisks as *p < 0.05, **p < 0.01 or ***p < 0.001.

From all three varieties and all four compartments, a total of 30 bacterial isolates were characterized for their SA production (details given in Supplementary Table 4), three of the SA-producing genera (some mentioned above) were identified from the phyllosphere of CLCuD-resistant FDH228 with the potential to see their utility as biocontrol agents. These are shown in Fig. 5a and are uncultured *Serratia spp*., *Bacillus spp*., and *Fictibacillus spp*. Through pot experiment, our results exhibit the efficacy of these SA-producing bacterial strains, particularly uncultured *Serratia spp*., as a biocontrol agent on the partially tolerant variety PFV-1 of *Gossypium hirsutum*. In the PFV-1 variety, the plants applied with *Serratia spp*. exhibited no CLCuD symptoms at 20 days post inoculation (DPI). The control group of PFV-1, on the other hand, displayed severe leaf curling and the dwarfing of the plant at 20 DPI (Fig. 5b, c). The average percentage of disease in the susceptible variety PFV-2 is shown in Fig. 5d, where the SA-producing strain, R1 uncultured *Serratia spp*., exhibited <10% CLCuD progression as compared to the PFV2.nA control group (25% at ~35–40 DPI). The percentage of disease in the partially tolerant variety PFV-1 is shown in Fig. 5e. The group treated with SA-producing strain R1 uncultured *Serratia spp*. and SA-producing strain R3 *Fictibacillus spp*. exhibited less than 10% CLCuD progression as compared to the PFV1.nA control group (40% at 40 DPI).

## Discussion

CLCuD is an acute disease of cotton, with economic losses reaching approximately 2 billion USD per annum in Pakistan^25^. In comparison, the major losses happened in the 1990s, when cotton yields were reduced by 75%^26^. In the past 5 years, Pakistan has fallen from the third largest^27^ to the fifth largest cotton-producing nation^28^, with CLCuD being the main contributor to this downfall. CLCuD is a whitefly-transmitted disease caused by the begomovirus. Pathogenic attacks constantly threaten plants, which pose a major global food security hazard. Over the past few years, extensive knowledge has been gathered from research on the effect of plant microbiome on fungal and bacterial pathogens. However, the role of the microbiome against viral pathogens remains unexplored. The endosphere and rhizosphere microbiome harbor potential microbes for suppressing plant diseases which can be exploited for combatting viral pathogens^29,30^. To our knowledge, this is one of the preliminary studies to investigate the phyllosphere and rhizospheric bacterial communities of CLCuV-infected susceptible, partially tolerant, and resistant cotton plants. It is vital to understand the basis of resistance in the CLCuV-resistant *G. arboreum* species, and here we present the microbiome angle of it.

The highest richness in taxonomy was observed in the RZ of the CLCuV-resistant FDH-228. *Pseudomonas* was predominantly found in the RZ of CLCuV-resistant *G. arboreum*. Rhizospheric microbiota in tomato plants is known to provide disease resistance against wilt caused by *Ralstonia solanacearum*^31^. Also, rhizobacteria belonging to the *Pseudomonadaceae, Bacillaceae, Solibacteraceae*, and *Cytophagaceae* families were reported to be prevalent in the rhizosphere of *Fusarium-*resistant bean cultivars^32^. No *bacillus* species were found in the leaf epiphytic region of CLCuV susceptible PFV-2. They are reported in the partially tolerant PFV-1 but most abundant in the resistant variety FDH-228.

The temporal and spatial dynamics of microbial communities play a pivotal role in understanding the underlying mechanisms of plant defense. Induced systemic resistance (ISR) is also activated in plants in an SA-dependent manner. *Pseudomonas aeruginosa* 7NSK2 failed to activate ISR in SA mutant tomato plants^33^. In the core microbiome analysis, *Pseudomonas* has been found in the LN and RN of *G. arboreum*. Endophytic bacteria such as *Bacillus, Pseudomonas*, and *Rhizobium* have been reported to confer pathogen resistance by the production of bioactive compounds^34–36^. *Pseudomonas*, which has been observed to be a key player in the CLCuD-resistant cotton variety’s microbiome, is known to synthesize SA from chorismate via the isochorismate synthase (ICS) and isochorismate pyruvate lyase^37,38^. *Pseudomonas* and *Bacillus* are known to be positively associated with CLCuV resistance, as exhibited in our GLLVM results. Other bacterial species known for endogenous SA production and enhancing plant host defenses by activation of SAR include *P. fluorescens* (strain CHA0), *P. aeruginosa* (7NSK2), and *Serratia marcescens* (strain 90-166)^39–41^.

The three identified SA-producing bacterial strains have been isolated from the CLCuD-resistant variety FDH-228. The application of bacterial strains from the resistant variety aids the susceptible *Gossypium hirsutum* in resisting disease compared to control plants. High SA levels during pathogen infection release NPR1 which is a key regulator of SAR^42,43^. Apart from pathogen exposure, ozone and UV-C light are known to induce SA synthesis in plants. SA has been reported to reduce disease incidence by activating SAR upon viral pathogenic attack^44^. Endophytic bacteria exhibit the potential to switch on plant defense responses and alter the rhizospheric microbiome. *Serratia marcescens strain* 90-166 predominantly found in the rhizosphere is known to provide resistance against fungal, bacterial, and viral pathogens^45^. *Bacillus megaterium* has also been reported for its role in rice spikelet rot disease suppression owing to the rhizospheric plant-microbe interactions and their correlation with plant resistance^46^.

The plant-pathogen and plant-microbe interactions represent how a plant immune system would react to a pathogen attack. Therefore, it is mandatory to explore the microbiome itself and the ‘microbiome under pathogenic attack,’ which reveals the answers to the overall success or failure of the plant-beneficial microbiome. Microbiota use may have a probiotic effect on plant functions, as the basis of plant defense is also a function of the plant genome and its microbial counterpart. Using the plant’s microbiome for suppressing begomoviral disease seems very promising. The SA producing bacterial isolates were applied as a biocontrol agent and strain R1 (uncultured *Serratia sp*.) performed as good as the positive control group where SA was applied as a protective regime in the susceptible variety PFV-2. The average percentage disease in partially tolerant variety PFV-1 remained less than 20% till 60 days post-viral inoculation (DPI) in plants applied with strain R1 (uncultured *Serratia sp*.), strain R3 (*Fictibacillus spp.)*, and SA respectively as compared to control (no application) with 40% disease progression at 60 DPI. Thus, the CLCuD-resistant variety-associated microbiome has the profound potential to resist disease under high whitefly attack in the highly susceptible *Gossypium hirsutum*.

High Salicylic Acid (SA) production in bacterial strains isolated from the phyllosphere of Cotton Leaf Curl Disease (CLCuD) resistant *Gossypium arboreum* exhibited the ability of these strains to activate the plant defense responses prior to viral pathogenic attack. Compared to the control, these strains proved efficient in suppressing viral disease in CLCuD-susceptible cotton species. The plants applied with *Serratia* and *Fictibacillus* strains in monoassociation experiments proved the most effective in CLCuD suppression. The microbiome analysis of *Gossypium hirsutum* and *Gossypium arboreum* has provided us with insights into the potential of the microbiome associated with the resistant species, and the biocontrol agents identified in this study have the potential to be utilized in intervention studies for field applications against CLCuD.

## Methods

### Study site and sample collection

Sampling was carried out at Four Brothers Research Farm (31.3991108° N,74.1743338° E) and the Greenhouse at Forman Christian College University, Lahore (31.5204° N, 74.3587° E). The region has a semi-arid climate with annual temperatures ranging from 13.5 to 40 °C and an average of 628.8 mm yearly rainfall. A total of 15 cotton plants were sampled across two CLCuD susceptible and resistant cotton species, therefore, 5 plants were sampled for each of the three selected varieties: *Gossypium hirsutum* varieties were PFV-2 (susceptible), PFV-1 (partially tolerant), and the selected *Gossypium arboreum* variety was FDH-228 (resistant). Plants heavily infested with whiteflies (containing CLCuV) were sampled at the vegetative stage (50 days after germination DAG). The susceptible variety exhibits disease symptoms at 25 DAG under heavy whitefly attack. The total samples amounted to 60 with five replicates for each of the four selected plant compartments [Leaf Epiphyte (LE), Leaf eNdophyte (LN), RhiZosphere (RZ), and Root eNdophyte (RN)].

### Beta satellite amplification for CLCuV confirmation

CLCuD-infected leaf tissues were taken from PFV-2 (susceptible), PFV-1 (partially tolerant), and FDH-228 (resistant) plants and were subjected to DNA extraction and beta-satellite amplification for CLCuV confirmation (Supplementary Fig. 1). Beta satellite DNA component of the CLCuD complex was amplified using the primers listed in Supplementary Table 1. For each reaction, 25 μL total volume was used with 12.5 μL Thermo Scientific DreamTaq Green PCR Master Mix (2X), 1 μL from 10 μM of each primer, 1 μL of MgCl_2_, 1 μL of total genomic DNA template (10 ng/μL), and remaining volume was made up with nuclease-free water. The PCR program was set as: 94 °C for 4 min (initial denaturation), followed by 30 cycles of 94 °C for 1 min (denaturation), 68 °C for 1 min (annealing), 72 °C for 1 min 30 s (extension) with a final extension of 10 min at 72 °C. Leaf enations/secondary leaf growth on the underside of the leaf as observed in Fig. 1a suggests that the plants were infected with the Cotton Leaf Curl Kokhran virus Burewala strain (CLCuKoV-Bu). This virus is associated with a recombinant betasatellite, termed as Cotton Leaf Curl Multan Betasatellite Burewala strain (CLCuMuB^Bur^). CLCuKoV-Bu is known to be prevalent in Lahore from where the sampling for this study was conducted^47^.

### Sample preparation and metagenomic DNA extraction

Rhizospheric soil (up to 3 mm around root area) and roots from CLCuD susceptible, partially tolerant, and resistant cotton plants (5 from each variety) were collected by gently shaking the roots of the plant to get rid of the bulk soil, and roots were stored in a 50 mL falcon tube. For leaf sample collection, leaves from the upper canopy showing CLCuD symptoms were taken for susceptible varieties since initial symptoms of CLCuD such as swelling and darkening of leaf veins, followed by a deep downward cupping are observed in the youngest leaves. For FDH-228 resistant plants, upper canopy leaves were sampled under a heavy whitefly attack to ensure CLCuV infestation. Samples were placed in an ice box until they were brought to the lab, and the standard protocol was followed for preparation before DNA extraction. For leaf epiphytes, the leaves were washed three times with 1X TE buffer containing 0.2% Triton X. The wash was collected and filtered through 0.2 µM sterile filter paper, which was used for DNA extraction. The leaves were washed with 70% ethanol, then with 3% bleach, and thoroughly with sterile distilled water (SDW) to eliminate leaf epiphytic bacteria. For leaf endophytes, 100 mg of leaf tissue was ground using a pestle and mortar in PBS buffer and collected in a falcon tube. Roots were sonicated in PBS buffer for 5 mins for rhizospheric soil to separate the closely adhered soil. The root was removed and used for DNA extraction of root endophytes. First, roots were washed with 70% ethanol, then 3% bleach, and several washings were given with SDW to eliminate rhizospheric bacteria. The sterilized root (100 mg) was macerated in PBS buffer using a pestle and mortar and was collected in a falcon tube. DNA for all four compartments (LE, LN, RZ, RN) was extracted using the FastDNA Spin Kit for Soil (MP Biomedicals) following the manufacturer’s guidelines. Samples were homogenized in the FastPrep instrument for 40 s at a speed setting 6.0. The DNA was eluted in 30 µL of elution buffer.

### 16S rRNA V3-V4 amplification and sequencing

A total number of 60 metagenomic DNA samples comprising the 4 plant compartments were sequenced on an Illumina MiSeq platform (Macrogen, Inc. Seoul, South Korea) using the primer set listed in Supplementary Table 1 with added Illumina adapter overhang nucleotide sequences for amplification of 16S rRNA hypervariable region V3-V4. Each reaction was performed in 25 μL total volume with 12.5 μL KAPA HiFi HotStart ReadyMix (Roche), one μL from 10 μM of each primer, one μL of each mPNA and pPNA blockers, two μL of metagenomic DNA template (10 ng/μL), and remaining volume was made up with nuclease-free water. The PCR program was set as follows 95 °C for 5 min (initial denaturation), followed by 35 cycles of 94 °C for 1 min (denaturation), 55 °C for 1 min (annealing), 72 °C for 1 min, 30 sec (extension) with a final extension of 10 min at 72 °C. PCR reactions were cleaned up with AMPure® XP beads^48^.

### Bioinformatics analysis

The 16S rRNA gene sequences for *n* = 59 samples [Study design: 3 varieties (FDH228, PFV-1, PFV-2) x 4 Compartments (LE, LN, RZ, RN) x 5 replicates; one PFV-1 LE was excluded due to poor DNA yield] were processed with the open-source bioinformatics pipeline QIIME2. The Deblur algorithm^49^ within the QIIME2 platform was used to recover 38,120 Amplicon Sequence Variants (ASVs). Briefly, the sequencing reads were imported to Qiime2 format, and were quality trimmed with a minimum Phred quality score of 20. Afterwards, we have used the qiime deblur denoise-other plugin with parameters –p-trim-length 280 –p-min-size 2 –p-min-reads 2 to generate ASVs. As a pre-processing step, the method also filters out any sequences that are not found in the reference SILVA SSU Ref NR database v138 which, is additionally used in qiime feature-classifier plugin to assign taxonomy to each ASV. This yielded a 59 (sample) X 38,120 (ASV) abundance table with summary statistics of sample reads as follows: [1^st^ Quartile:7,979; Median:15,522; Mean: 14;565; 3^rd^ Quartile:21,387; and Maximum: 27,839]. Afterwards qiime phylogeny align-to-tree-mafft-fasttree generated the rooted phylogenetic tree of all the ASVs. The biom file for the ASVs was generated by combining the abundance table with taxonomy information using biom utility available in qiime2 workflow. PICRUSt2 algorithm as a QIIME2 plugin was used on the ASVs to predict the functional abundance of microbial communities associated with the CLCuD susceptible and resistant cotton microbiome. We used PICRUSt2^50^ and its qiime2 plugin [https://github.com/gavinmdouglas/q2-picrust2] using the parameters –p-hsp-method pic –p-max-nsti 2 in qiime picrust2 full-pipeline to find KEGG enzymes and MetaCyc pathway predictions. For ensuing statistical analysis, any sample having <5000 total reads was dropped, excluding further contaminants based on taxonomy (Chloroplast, Mitochondria, and ASVs unassigned at Phylum level).

### Statistical analysis

Statistical analyses were performed in R using the combined data generated from the bioinformatics as well as meta data associated with the study. The R’s Vegan package was used for the analysis of alpha diversity of all tables. For alpha diversity, the indices used were (i) rarefied richness – the number of expected features in a rarefied sample (to the minimum library size, and (ii) Shannon entropy – an index that takes into account both richness and diversity to provide a measurement of community balance. For beta diversity, the dissimilarity in species community composition between pairwise comparisons of bacterial communities were represented in Principal Coordinate Analysis (PCoA) ordination plots by using three different distance metrices in Vegan ’s cmdscale function: (i) Bray–Curtis, which considers the species abundance count; (ii) Unweighted Unifrac, which considers the phylogenetic distance between the branch lengths of ASVs observed in different samples (implemented in the phyloseq package^51^, and (iii) Hierarchical Meta-Storms (HMS)^52^, a functional beta diversity distance which collapses the metabolic pathways at the observed KOs level by considering BRITE pathways as a set of reference pathways, and then propagating the abundances upward for these pathways in a multi-level pathway hierarchy to give a weighted dissimilarity measure. To see if beta diversity is statistically significant between the groups, we have used Vegan’s adonis for analysis of variance using distance matrices (BrayCurtis/Unweighted Unifrac/Hierarchical Meta-Storms) This function, henceforth referred to as PERMANOVA, fits linear models to distance matrices and uses a permutation test with pseudo-F ratios and gives an *R*^2^ value for each covariate defined as percentage variability in community structure explained by that covariate.

To find the core microbiome, we have used R’s microbiome package^53^ which offers a two-dimensional representation of core communities by taking an assumed initial prevalence (50% presence of a microbe in the total number of samples) and then calculating the detection limit in terms of abundances to differentiate between low-abundance core microbiome versus high-abundance core microbiome. The initial prevalence threshold was set to 50%. In addition, we have generated taxa bar plots of the top 20 most abundant phyla at appropriate taxonomic levels to give an indication of how dominant phyla change between conditions.

To see the relationship between different conditions (cotton plant varieties and compartments) and the minimal subset of microbes that can explain them, we used the variable selection approach where through penalized regression on the set of all pairwise log-ratios we identify two disjoint subsets of microbes, those that are positively associated, and those that are negatively associated with the covariate of interest. Briefly, we used the CODA-LASSO approach^54^ where the abundance of individual covariate *y*_i_ is modeled as ${\it{y}}_{\it{i}} = \beta _0 + \beta _1\;{{{\mathrm{log}}}}\left( {{\it{x}}_{1{\it{i}}}} \right) + \ldots + \beta _{\it{j}}\;\log \left( {{\it{x}}_{{\it{ji}}}} \right){\it{ \in }}_{\it{i}}$ (for *i*-th sample and *j*-th species/function, with *x*_*ji*_ being the microbe abundance) with the constraint $\mathop {\sum}\nolimits_{k \ge 1} {\beta _k = 0}$ (i.e., all *β*-coefficients sum up to 0), and these regression coefficients ***β*** = (*β*_0_,…, *β*_*j*_) are estimated to minimize $\mathop {\sum}\nolimits_{i = 1}^n {\left( {y_i - \beta _0 - \beta _1\log \left( {x_{1i}} \right) - \ldots - \beta _j\log \left( {x_{ji}} \right)} \right)^2 + \lambda \mathop {\sum}\nolimits_{k \ge 1} {\left| {\beta _k} \right|} }$ subject to $\mathop {\sum}\nolimits_{k \ge 1} {\beta _k = 0}$ (using a soft thresholding and projection algorithm) for *n* samples. Here, *λ* is the penalization parameter in Lasso shrinkage terms $\lambda \mathop{\sum}\nolimits_{k \ge 1} {\left| {\beta _k} \right|}$ which forces some of the ***β***-coefficients to go zero, particularly those that do not have a relationship with the covariates and serves as a means to do variable selection. The non-zero ***β***-coefficients are then divided into two groups, those that are positively associated with the environmental covariate, and those that are negatively associated with the environmental covariate, respectively. For this purpose, we used coda glmnet () function from R’s coda4microbiome package^55^. We have used the top 100 most abundant genera/functions in the CODA-LASSO model. For visualizing the expression of microbes/functions selected from the procedure, we have used TSS + CLR normalization (Total Sum Scaling followed by Centralized Log Ratio).

To find the relationship between microbial communities and sources of variation (Cotton Species, Cotton Variety, CLCuV_Susceptibility, and Compartment), we have used Generalised Linear Latent Variable Model (GLLVM)^56^ which extends the basic generalized linear model that regresses the mean abundances *μ*_*ij*_ (for *i*-th sample and *j*-th microbe) of individual microbes against environmental covariates *x*_*i*_ as above by incorporating latent variables *u*_*i*_ as $g(\mu _{ij}) = n_{ij} = \alpha _i + \beta _{0j} + {{{\boldsymbol{x}}}}_i^T\beta _j + {{{\boldsymbol{u}}}}_i^T\theta _j$, where ***β***_*j*_ are the microbe specific coefficients associated with individual covariate (a 95% confidence interval of these whether positive or negative, and not crossing 0 boundary gives directionality with the interpretation that an increase or decrease in that particular covariate causes an increase or decrease in the abundance of the microbe), and ***θ***_*j*_ are the corresponding coefficients associated with latent variable. *β*_0*j*_ are microbes’ specific intercepts, whilst *α*_*i*_ are optional sample effects which can either be chosen as fixed effects or random effects. To model the distribution of individual microbes, we have used Negative Binomial distribution. Additionally, the approximation to the log-likelihood is done through Laplace approximation (LA) with final sets of parameters in glvmm function being family = ‘negative.binomial’, method = “LA”, and starting.val = ‘zero’ that seemed to fit well. This, we did for top 100 most abundant genera in our datasets. In addition, the factor loadings ***θ***_*j*_ store correlations of microbes with the residual covariance matrix ***Σ***=***ΓΓ***^*T*^ where ***Γ***=[*θ*_1_…*θ*_*m*_] for *m* latent variables. This residual covariance matrix gave co-occurrence relationship between microbes that are not explained by environmental covariates as above.

All figures in this study were generated using R’s ggplot2 package. For alpha diversity we have used ANOVA, and where two categories are significantly different, following annotations are used to denote significance: ‘***’ (*p* ≤ 0.001, ‘**’ (*p* ≤ 0.01), ‘*’ (*p* ≤ 0.05), and ‘.’ (*p* ≤ 0.1).

### Isolation and characterization of culturable microorganisms

Microorganisms from all four plant compartments (LE, LN, RZ, RN) from the three selected CLCuV-infested cotton varieties were isolated on Tryptic Soy Agar (TSA) and Reasoner’s 2 A (R2A) media, followed by the plate count technique.

Cell morphology was studied under light microscope. Gram staining^57^ was used to differentiate between Gram-positive and Gram-negative bacteria. Motility of bacterial isolates was observed under light microscope. Sterilized distilled water was used to prepare bacterial suspension. A drop of suspension was added to the glass slide and observed under the microscope.

Quick test strips (QTS) 24 bacterial identification kits (DESTO Laboratories, Karachi Pakistan) were used to detect enzymes and carbon source utilization pattern. Bacterial suspension was prepared in sterilized falcon tubes from freshly streaked colonies. Incubation box of QTS strips were partially filled with water to make the inside environment of QTS-24 strips moist. QTS-24 strips were placed in the incubation boxes. All the cups were partially filled with except cups of UREA and MOT (Cell Motility) that were completely filled with bacterial suspension. A gelatin disc was placed in cup labeled with GEL in each strip. Sterilized liquid paraffin (Mineral oil) was poured in cup labeled with ADH (Arginine deaminases) and H_2_S for an-aerobiosis. Inoculated QTS-24 strips were incubated at 28 °C. After 24 h incubation, strips were examined, and the results were interpreted according to the manufacturer’s manual. For TDA (Tryptophan deaminase) test, 1–2 drops of 10% ferric chloride 12 was added in TDA labeled cup. 0.8% sulfonic acid and 0.5% alpha-naphthylamine in 5 N acetic acid was used for the detection of nitrate reductase test. For VP test, 40% KOH and 5% ethanolic solution of alpha-naphthol was added in VP labeled cup and incubated at 37 °C for 10 min. For indole test, single drop of Kovac’s reagent was added into cup labeled as IND (indole-3-acetic acid).

### Screening bacterial isolates for phytohormone production

IAA production was estimated by colorimetric method using the Salkowski reagent^58^. The bacterial strains were grown in Luria Bertani (LB) broth containing tryptophan (100 mg/10 ml) for 7-10 days. Then, the cells were harvested at 10,000 rpm for 15 minutes. The pellet was discarded. Salkowski reagent (4 ml) was added to 2 ml of sample and was incubated at 28 °C for 30 minutes. The absorbance for samples was noted in triplicates at 530 nm.

The sample preparation was carried out for each bacterial isolate for salicylic acid production assessment using HPLC analysis^59^. Each bacterial strain was seeded into a plastic tube containing King’s B Broth (20 ml) from a single colony on a TSA plate grown for 24 h at 28 °C. SA was extracted after 48 h from the stationary phase. At that time, bacterial cultures contain 10^10^–10^11^ cells. The liquid culture was centrifuged at 2800 g for 20 mins at 4 °C. The supernatant was acidified to pH = 2 with 1 N HCl. The solution was filtered through 0.22 µm nylon membrane, partitioned twice with 2 ml CHCl_3_, and dried under a nitrogen stream at 4 °C. These samples were subjected to HPLC, carried out on a Waters^TM^ HPLC System where molecular-grade salicylic acid was used as a standard. Methanol in sodium acetate buffer was used as a mobile phase.

### 16S rRNA Identification of SA-producing bacterial strains

Bacterial colonies were directly used for amplification of the V3-V4 region using the primer pair listed in Supplementary Table 1. The bacterial colony was directly placed in the PCR mixture. The PCR profile used for amplification of V3-V4 region: 95 °C for 5 min, 35 cycles of 94 °C for 1 min, 55 °C for 1 min, 72 °C for 1min30sec and final extension of 72 °C for 10 min. Water was used as a negative control. PCR products were analyzed on 2% agarose gel.

Amplified PCR products of the salicylic-producing strains were sequenced using the commercial service of Sanger Sequencing at Macrogen (Seoul, Korea). The strains were identified using the V3-V4 sequence of the 16S rRNA gene using BLASTN search on DDBJ/NCBI servers.

### CLCuD disease incidence assay

#### Determination of soil properties

Before the pot experiments, the soil used in the study was sent to PCSIR Laboratories, Pakistan, for soil texture and chemical analysis. The following parameters were determined: pH, EC (µScm-1), organic matter, N, P, K, C, Mg, Na, and Mn, with results in Supplementary Table 5. Autoclaved soil was used for pot experiments in the climate control room.

#### Pot experiment

The disease incidence assay was carried out at Forman Christian College (A Chartered University), Lahore, Pakistan. The three SA-producing candidate strains (R1, R2, R3) were selected for application to the CLCuD susceptible (PFV-2) and partially tolerant (PFV-1) *Gossypium hirsutum* varieties in monoassociation as well as a synthetic consortium. For each genotype, a seven-day-old seedling was inoculated with bacterial suspension/synthetic consortium at 1 ×10^8^ cfu/g via soil drench application.

Exogenous SA (0.4 g/L) was applied as a foliar spray for the positive control group. The application was carried out in the climate control room. Plants were shifted to the greenhouse at 21 days post application (28 DAG) for viral inoculation. For each treatment group, a total of 30 replicates were taken. Data was collected for percentage disease and disease severity index calculation till 60 days post inoculation (DPI)^60^.

#### Disease index statistics

Disease index (DI) statistics were based on a scale of 0–6.0 (Supplementary Table 2). In the disease severity index, 6 is the highest disease score. The average percentage disease and disease severity index were calculated using the following formulae:

1 $${{{\mathrm{Percentage}}}}\,{{{\mathrm{disease}}}}\left( \% \right) = {{{\mathrm{Diseased}}}}\,{{{\mathrm{leaves/Total}}}}\,{{{\mathrm{leaves}}}} \times 100$$

2 $${{{\mathrm{Disease}}}}\,{{{\mathrm{Severity}}}}\,{{{\mathrm{index}}}}\left( {{{{\mathrm{DSI}}}}} \right) = {{{\mathrm{Average}}}}\,{{{\mathrm{Percentage}}}}\,{{{\mathrm{Disease/100}}}} \times {{{\mathrm{6}}}}$$

### Statistical analysis

SPSS software was used for data analysis, and where Tukey’s HSD post hoc test was performed with a significance level (*p* < 0.05).

### Supplementary information

Supplementary_Materials.pdf

Supplementary_Data_Table_1.csv

## Data Availability

The raw sequence files supporting the results of this article are available in the European Nucleotide Archive under the project accession number PRJEB67645 with details of the samples provided in Supplementary_Data_Table_1.csv.

## Code availability

Some of the code used to generate the results are part of microbiomeSeq (https://github.com/umerijaz/microbiomeSeq), whilst other codes are available at http://userweb.eng.gla.ac.uk/umer.ijaz#bioinformatics.
