# Supplementary material for: Cotton microbiome profiling and Cotton Leaf Curl Disease (CLCuD) suppression through microbial consortia associated with Gossypium arboreum
Source: NPJ Biofilms Microbiomes. 2023 Dec 14;9:100. doi: 10.1038/s41522-023-00470-9 (PMC10721634; doi:10.1038/s41522-023-00470-9)
Supplement: Supplementary file 1 — Supplementary_Materials.pdf [file 41522_2023_470_MOESM1_ESM.pdf]

e-Supplementary Methods

Title: Cotton Microbiome Profiling and Cotton Leaf Curl Disease (CLCuD) Suppression through Microbial Consortia associated with *Gossypium arboreum*

Rhea Aqueel [a,b], Ayesha Badar [a], Nazish Roy [a], Qandeel Mushtaq [a], Aimen Fatima Ali [a], Aftab Bashir [a], Umer Zeeshan Ijaz [b,c,d*], Kauser Abdulla Malik [a,e*]

[a]Kauser Abdulla Malik School of Life Sciences, Forman Christian College (A Chartered University), Ferozepur Road, Lahore 54600, Pakistan

[b]Water & Environment Research Group, University of Glasgow, Mazumdar-Shaw Advanced Research Centre, Glasgow G11 6EW, United Kingdom

[c]National University of Ireland, Galway, University Road, Galway, H91 TK33, Ireland

[d]Department of Molecular and Clinical Cancer Medicine, University of Liverpool, Liverpool L69 7BE, United Kingdom

[e]Pakistan Academy of Sciences, Islamabad

*** Joint corresponding authors (both authors jointly directed this work) umer.ijaz@glasgow.ac.uk (http://userweb.eng.gla.ac.uk/umer.ijaz) kausermalik@fccollege.edu.pk**

Supplementary Table 1. Primers used in the study.

Target Gene	Primer Sequence	Amplicon Size	Reference
**16S rRNA V3-V4 with Illumina adapter hang sequences**	341F (5′-TCGTCGGCAGCGTCAGATGTGTATAAGAGACAGCCTACGGGNGGCWGCAG-3′)<br>805R (5′-GTCTCGTGGGCTCGGAGATGTGTATAAGAGACAGGACTACHVGGGTATCTAATCC-3′)	~467 bp	(1)
**16S rRNA V3-V4 for Sanger Sequencing**	341F (5'- CCTACGGGNGGCWGCAG -3')<br>805R (5'- GACTACHVGGGTATCTAATCC -3')	~444bp	(1)
**Beta-Satellite DNA**	F-5’- GGTACCACTACGCTACGCAGCAGCC-3’<br>R-5’- GGTACCTACCCTCCCAGGGGTACAC-3’	1350bp	(2)

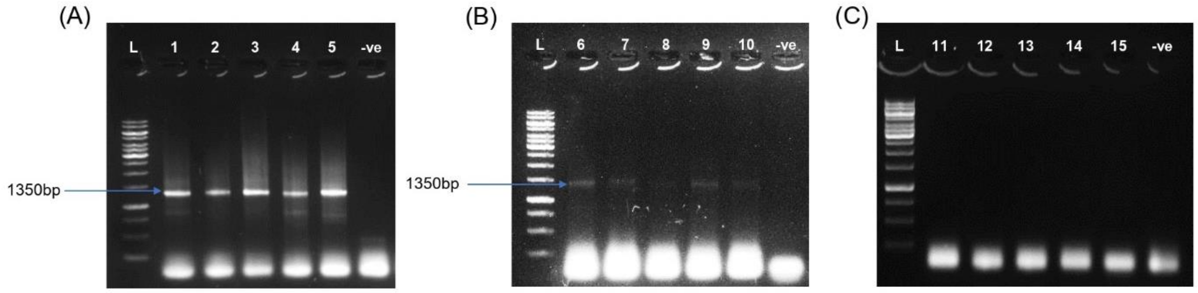

Supplementary Figure 1. PCR Amplification of beta satellite DNA of CLCuV Infected Cotton Leaf Samples run on 1% Agarose gel. (A) L = 1kb DNA ladder; Samples 1-5 (PFV-2 susceptible *G.hirsutum*); -ve (negative control with water) (B) L = 1kb DNA ladder; Samples 6-10 (PFV-1 partially tolerant *G.hirsutum*); -ve (negative control with water) (C) L = 1kb DNA ladder; Samples 11-15 (FDH-228 resistant *G.arboreum*); -ve (negative control with water)

Supplementary Table 2 - Disease Severity Index Scale for Cotton Leaf Curl Disease (CLCuD) as per [3].

**Disease Severity Index**	**Symptoms**
**0**	Complete absence of CLCuD symptoms and virus is undetectable in plant tissues using PCR.
**1**	Complete absence of symptoms, but virus can be detected in plant tissues using PCR. Thickening of veins or only presence of leaf enations on one or few leaves of upper canopy.
**2**	Thickening of small group of veins, no leaf curling, no reduction in leaf size and boll setting. No leaf curling observed.
**3**	Thickening of all veins, minor leaf curling and minor reduction in leaf size but no reduction in boll setting.
**4**	Severe vein thickening in leaves, moderate leaf curling followed by minor deformity of internodes and minor reduction in leaf size and boll setting.
**5**	Severe vein thickening, moderate leaf curling and deformity of internodes with moderate reduction in leaf size and boll setting followed by moderate stunting of the cotton plant.
**6**	Severe vein thickening, leaf curling, reduction in leaf size, deformed internodes and stunting of the plant with no or few boll setting 6 >50 Highly susceptible

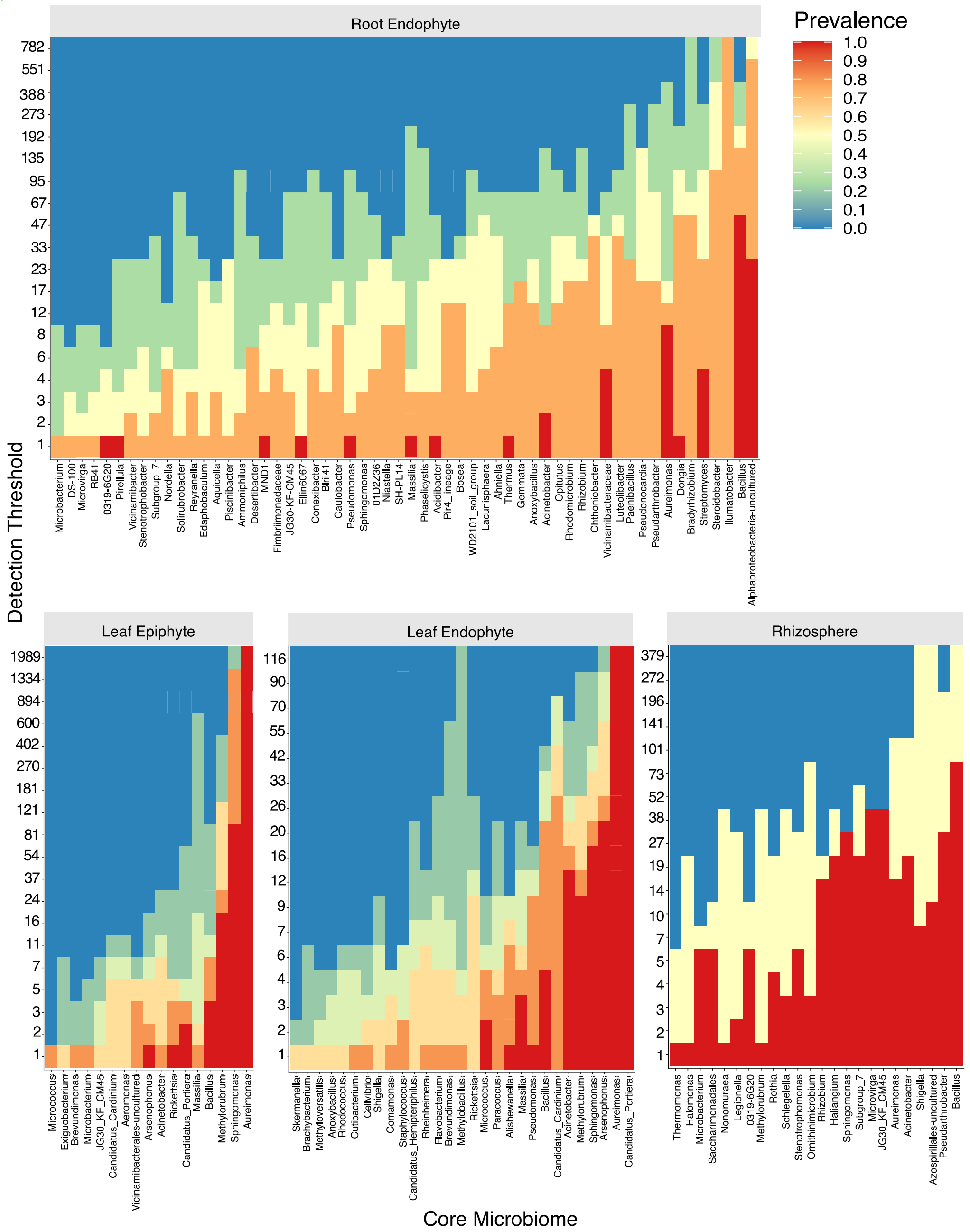

Supplementary Figure 2. Core microbiome analysis *Gossypium hirsutum* PFV-2: Heatmaps of the different meta-sample groups at genus level (Root Endophyte, Rhizosphere, Leaf Epiphyte, and Leaf Endophyte) derived from this study with a minimum prevalence of 50% and microbes sorted as left (low abundance) to right (high abundant) core microbiome.

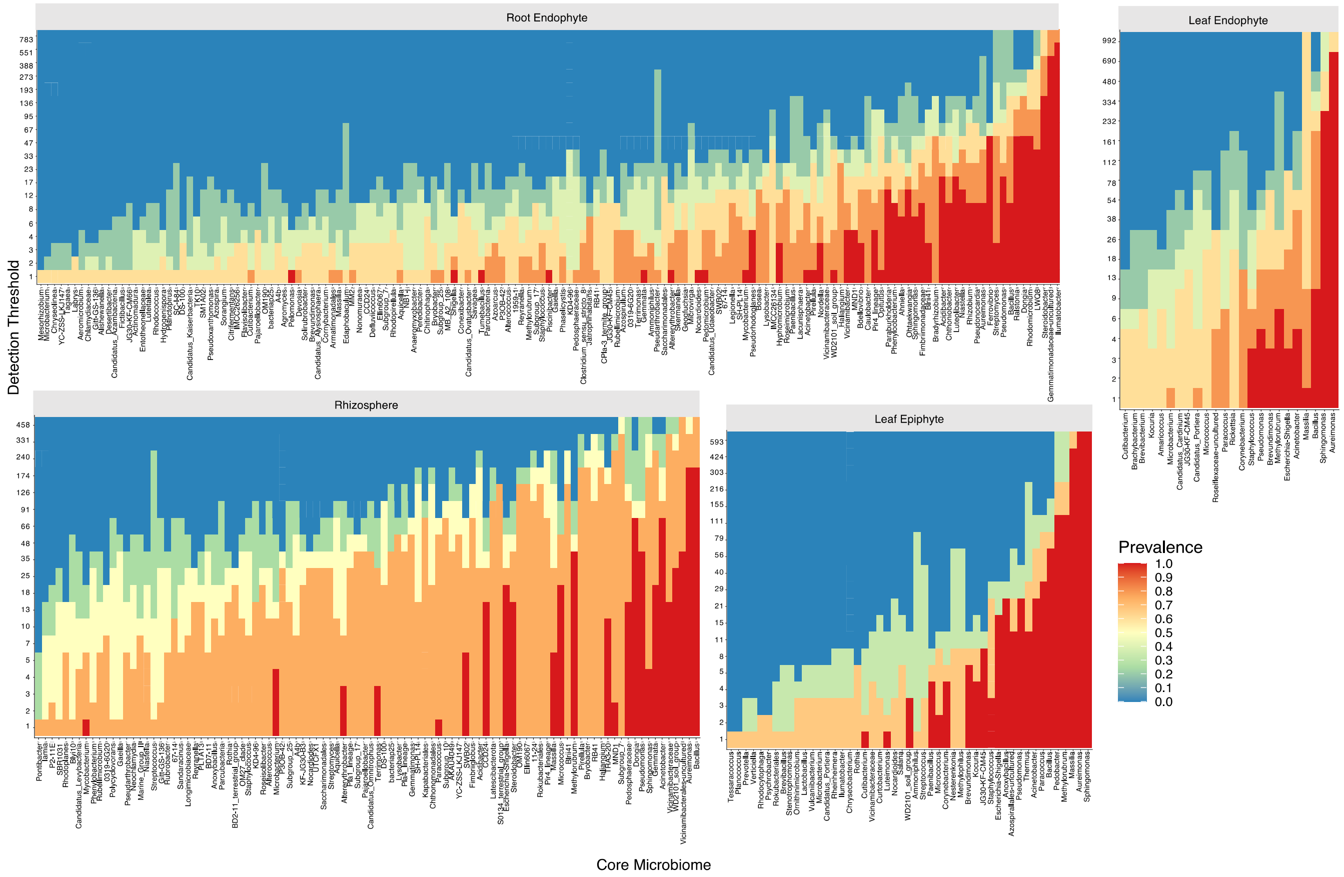

Supplementary Figure 3. Core microbiome analysis of *Gossypium hirsutum* PFV1: Heatmaps of the different meta-sample groups at genus level I (Root Endophyte, Rhizosphere, Leaf Epiphyte, and Leaf Endophyte) derived from this study with a minimum prevalence of 50% and microbes sorted as left (low abundance) to right (high abundant) core microbiome.

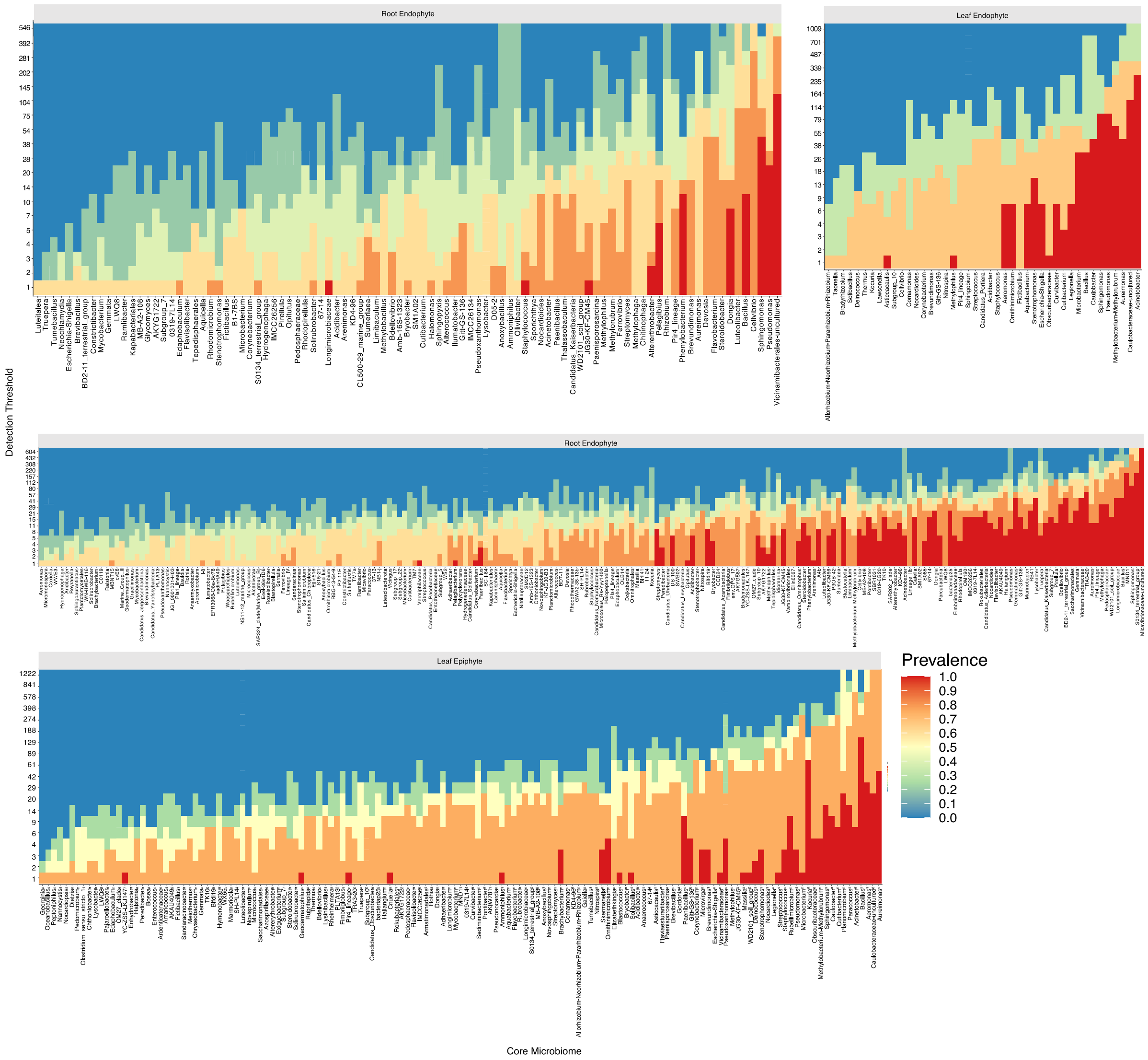

Supplementary Figure 4. Core microbiome analysis of *Gossypium arboreum FDH228*: Heatmaps of the different meta-sample groups at genus level I (Root Endophyte, Rhizosphere, Leaf Epiphyte, and Leaf Endophyte) derived from this study with a minimum prevalence of 50% and microbes sorted as left (low abundance) to right (high abundant) core microbiome.

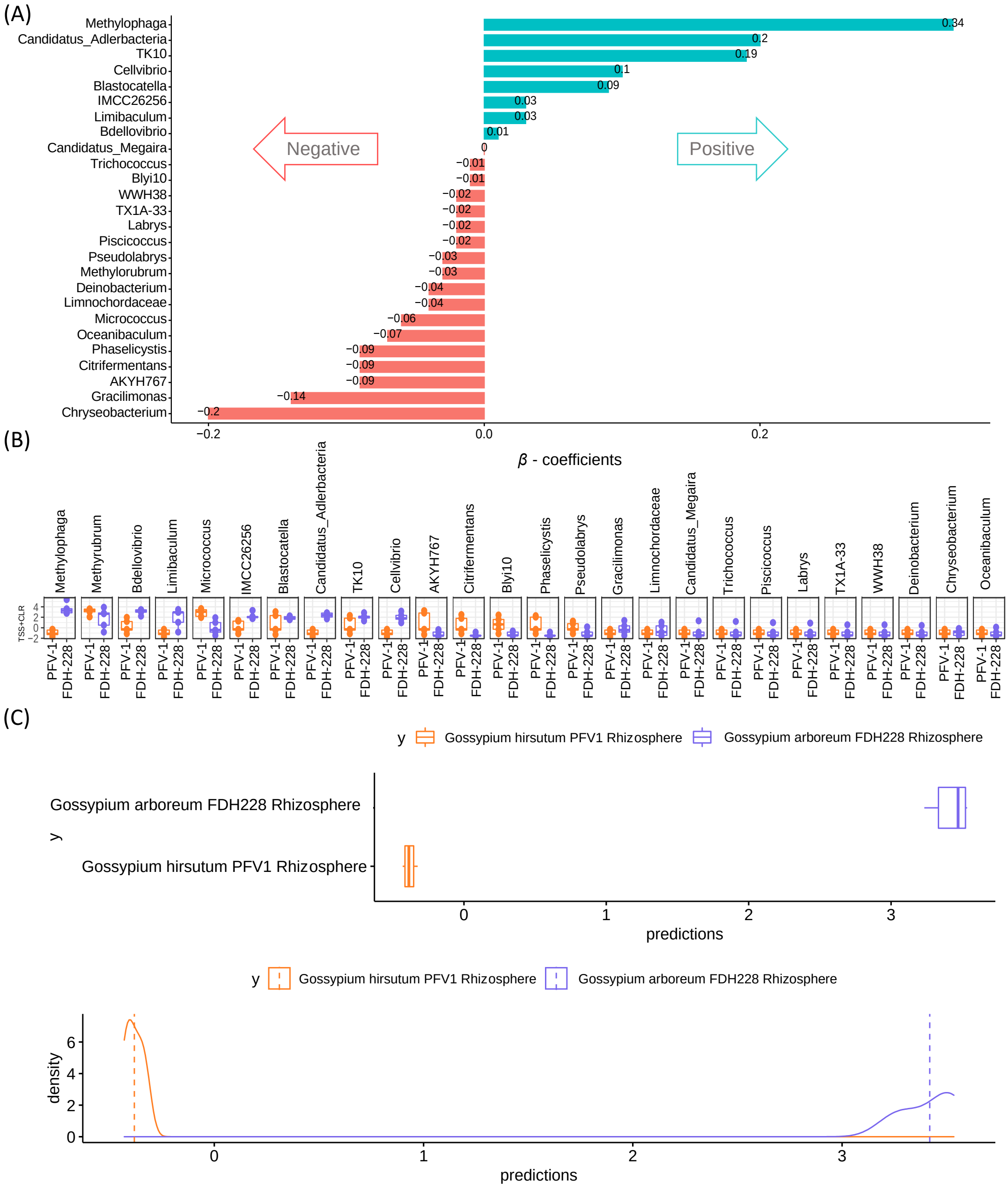

Supplementary Figure 5. CODA LASSO regression for *Gossypium hirsutum* PFV1 and *Gossypium arboreum* FDH228 (Rhizosphere) for taxonomic abundance at Genus level
A) β −coefficients returned from CODA-LASSO procedure as two disjoint sets, with those that are associated with FDH228 (Positive), and those that are associated with PFV-1 (Negative); B) Expression levels of microbes selected from the procedure; and C) The density plot returned from the CODA-LASSO segregates the two groups provides a graphical assessment of the classification accuracy (top: true; bottom: predicted from the procedure).

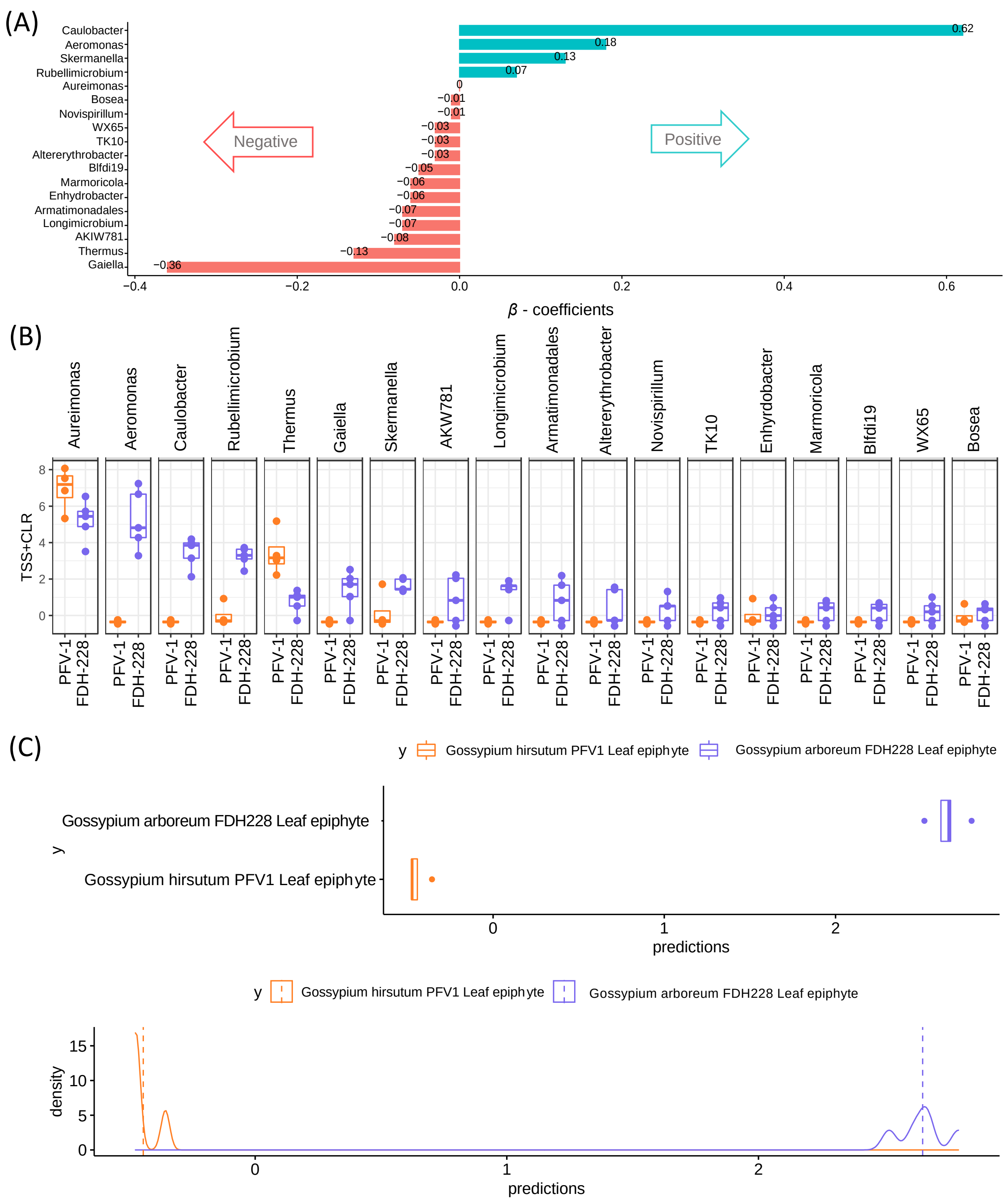

Supplementary Figure 6. CODA LASSO regression for *Gossypium hirsutum* PFV1 and *Gossypium arboreum* FDH228 (Leaf epiphyte) for taxonomic abundance at Genus level
A) β −coefficients returned from CODA-LASSO procedure as two disjoint sets, with those that are associated with FH228 (Positive), and those that are associated with PFV-1 (Negative); B) Expression levels of microbes selected from the procedure; and C) The density plot returned from the CODA-LASSO segregates the two groups provides a graphical assessment of the classification accuracy (top: true; bottom: predicted from the procedure).

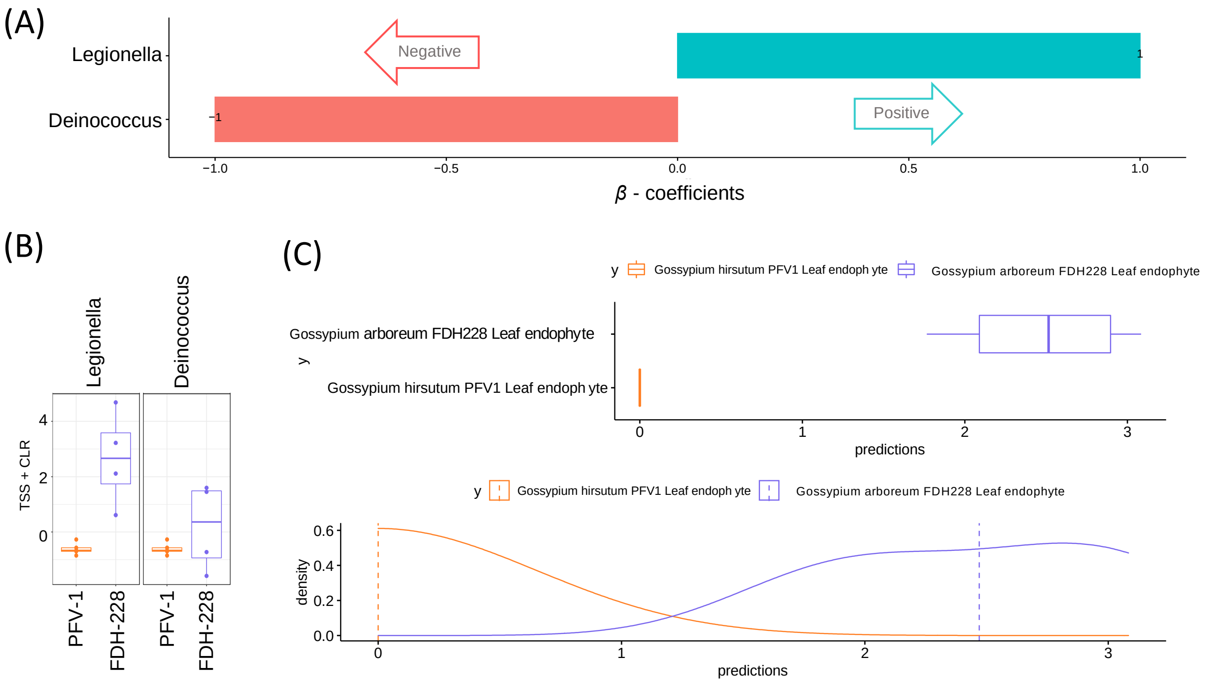

Supplementary Figure 7. CODA LASSO regression for *Gossypium hirsutum* PFV1 and *Gossypium arboreum* FDH228 (Leaf endophyte) for taxonomic abundance at Genus level A) β –coefficients returned from CODA-LASSO procedure as two disjoint sets, with those that are associated with FDH228 (Positive), and those that are associated with PFV-1 (Negative); B) Expression levels of microbes selected from the procedure; and C) The density plot returned from the CODA-LASSO segregates the two groups provides a graphical assessment of the classification accuracy (top: true; bottom: predicted from the procedure).

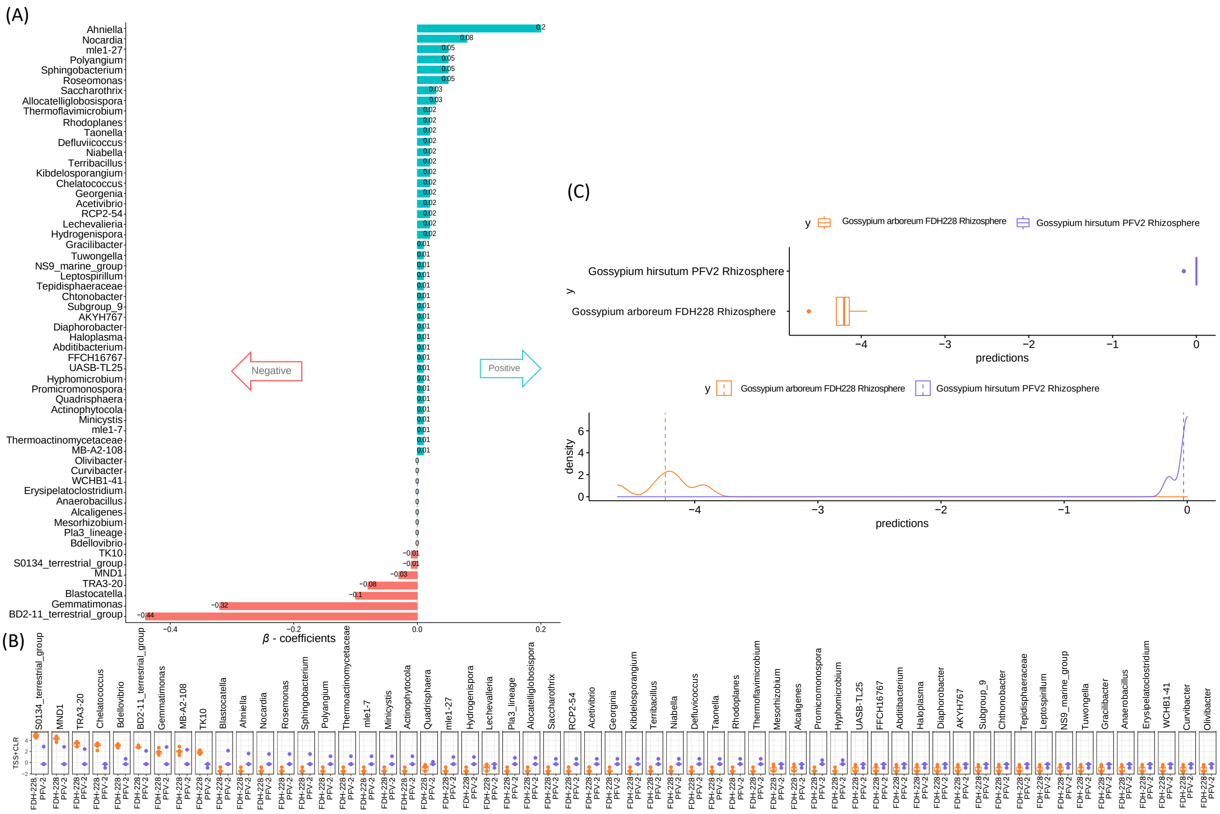

Supplementary Figure 8. CODA LASSO regression for *Gossypium arboreum FDH-228* and *Gossypium hirsutum* PFV-2 (Rhizosphere) for taxonomic abundance at Genus level
A) β −coefficients returned from CODA-LASSO procedure as two disjoint sets (those that are positively related, and those that are negatively related with the temperature) B) The density plot returned from the CODA-LASSO segregates the two groups provides a graphical assessment of the classification accuracy (top: true; bottom: predicted from the procedure); C) Expression levels of microbes selected from the procedure.

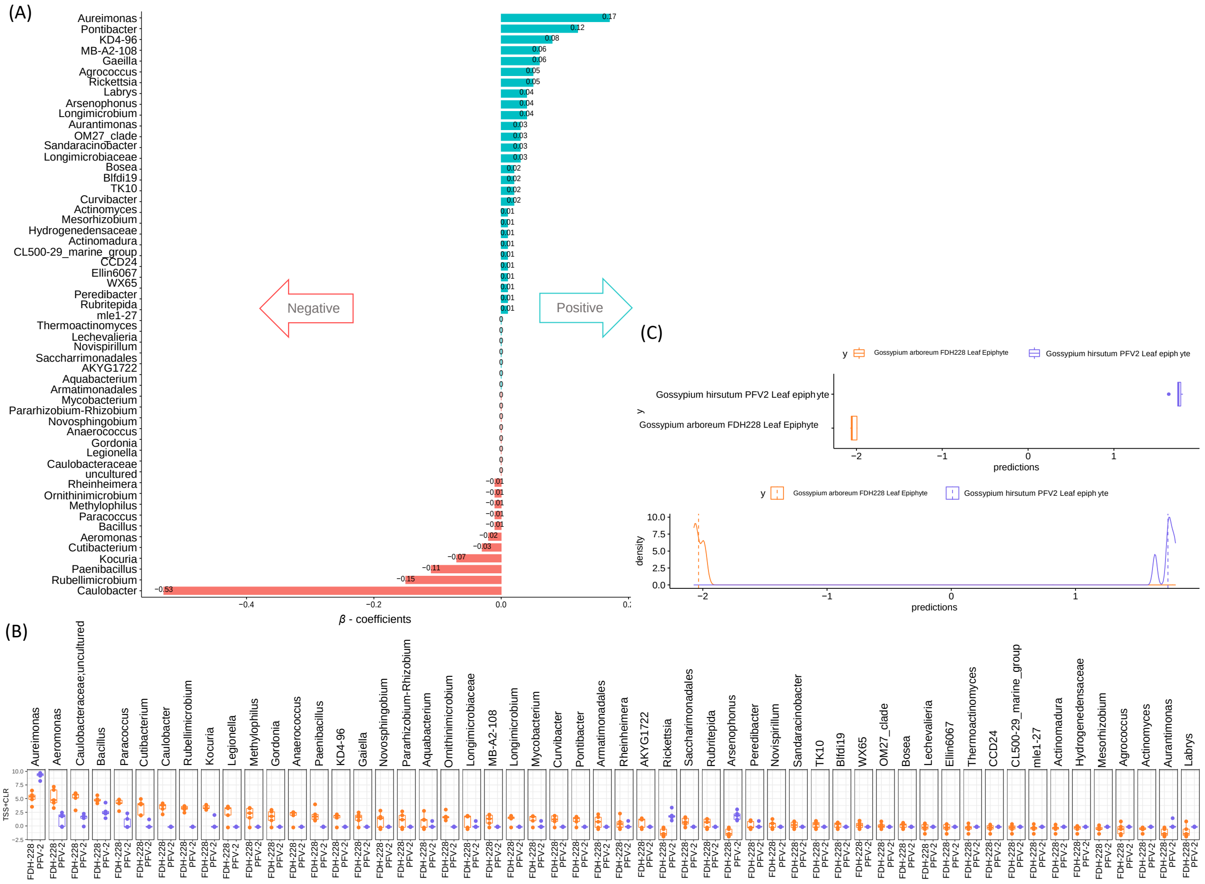

Supplementary Figure 9. CODA LASSO regression for *Gossypium arboreum FDH-228* and *Gossypium hirsutum* PFV-2 (Leaf Epiphyte) for taxonomic abundance at Genus level A) β –coefficients returned from CODA-LASSO procedure as two disjoint sets (those that are positively related, and those that are negatively related with the temperature) B) The density plot returned from the CODA-LASSO segregates the two groups provides a graphical assessment of the classification accuracy (top: true; bottom: predicted from the procedure); C) Expression levels of microbes selected from the procedure.

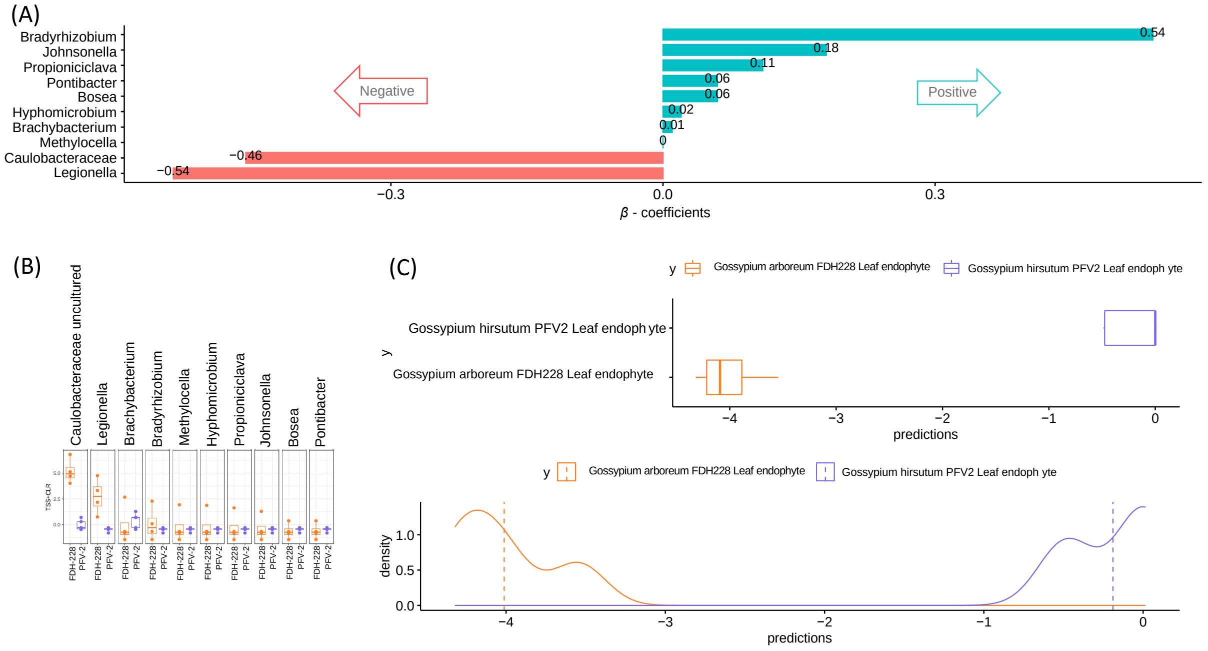

Supplementary Figure 10. CODA LASSO regression for *Gossypium arboreum FDH-228* and *Gossypium hirsutum* PFV-2 (Leaf Endophyte) for taxonomic abundance at Genus level A) β −coefficients returned from CODA-LASSO procedure as two disjoint sets (those that are positively related, and those that are negatively related with the temperature) B) The density plot returned from the CODA-LASSO segregates the two groups provides a graphical assessment of the classification accuracy (top: true; bottom: predicted from the procedure); C) Expression levels of microbes selected from the procedure.

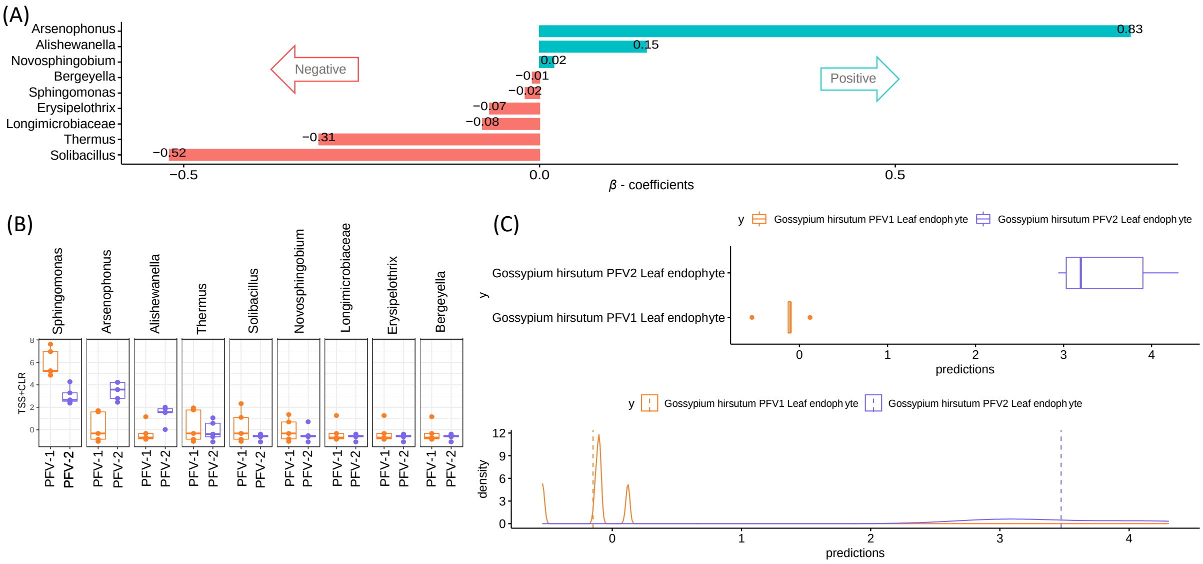

Supplementary Figure 11. CODA LASSO regression for *Gossypium hirsutum* PFV-2 and *Gossypium hirsutum* PFV-1 (Leaf Endophyte) A) β −coefficients returned from CODA-LASSO procedure as two disjoint sets (those that are positively related, and those that are negatively related with the temperature) B) The density plot returned from the CODA-LASSO segregates the two groups provides a graphical assessment of the classification accuracy (top: true; bottom: predicted from the procedure); C) Expression levels of microbes selected from the procedure.

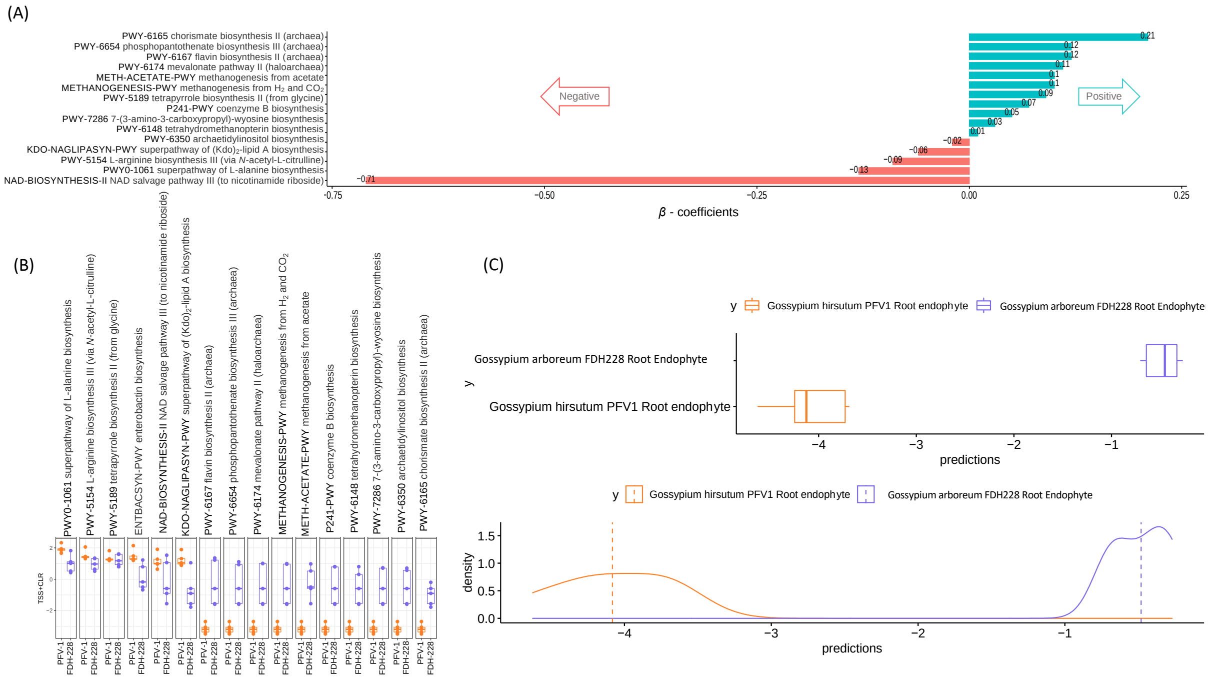

Supplementary Figure 12. CODA LASSO regression for *Gossypium hirsutum* PFV-1 and *Gossypium arboreum FDH-228* (Root Endophyte) for MetaCyc pathways A) β −coefficients returned from CODA-LASSO procedure as two disjoint sets (those that are positively related, and those that are negatively related with the temperature) B) The density plot returned from the CODA-LASSO segregates the two groups provides a graphical assessment of the classification accuracy (top: true; bottom: predicted from the procedure); C) Expression levels of microbes selected from the procedure.

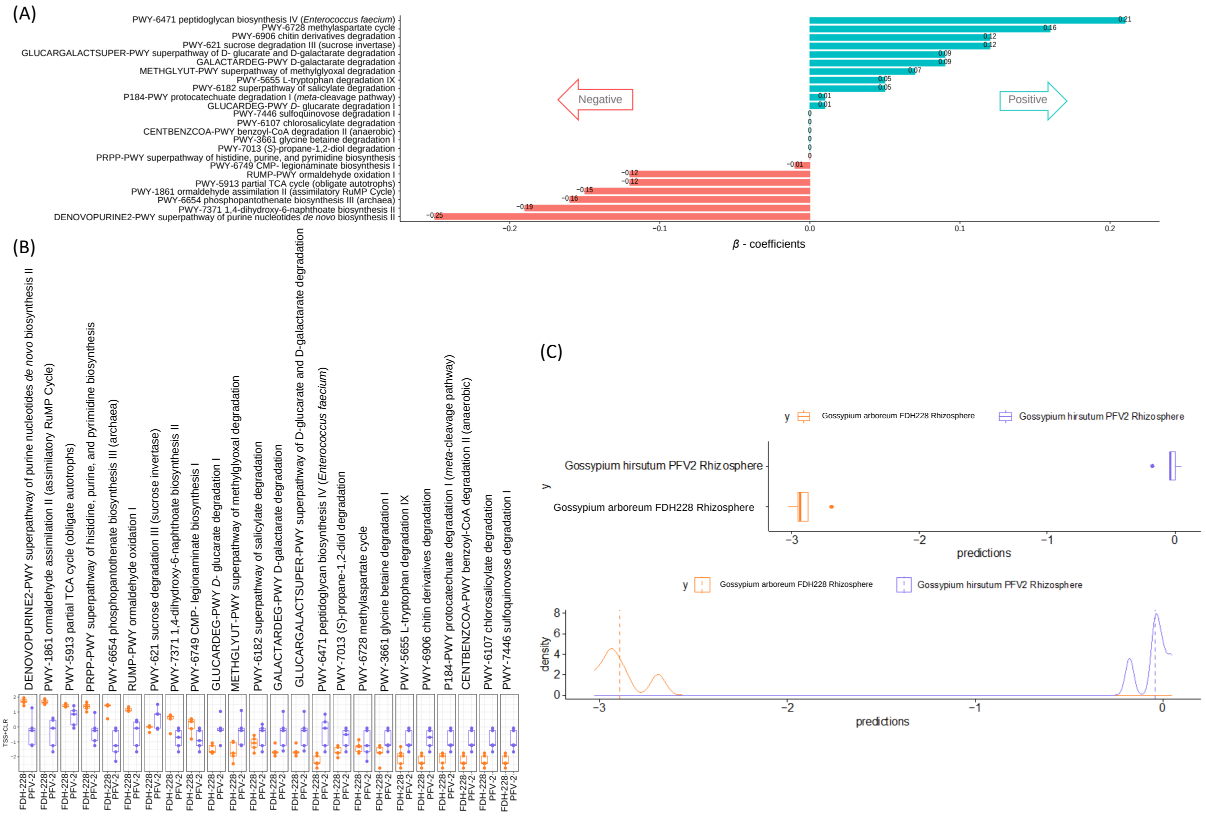

Supplementary Figure 13. CODA LASSO regression for *Gossypium hirsutum* PFV-2 and *Gossypium arboreum FDH-228* (Rhizosphere) for MetaCyc pathways A) β –coefficients returned from CODA-LASSO procedure as two disjoint sets (those that are positively related, and those that are negatively related with the temperature) B) The density plot returned from the CODA-LASSO segregates the two groups provides a graphical assessment of the classification accuracy (top: true; bottom: predicted from the procedure); C) Expression levels of microbes selected from the procedure.

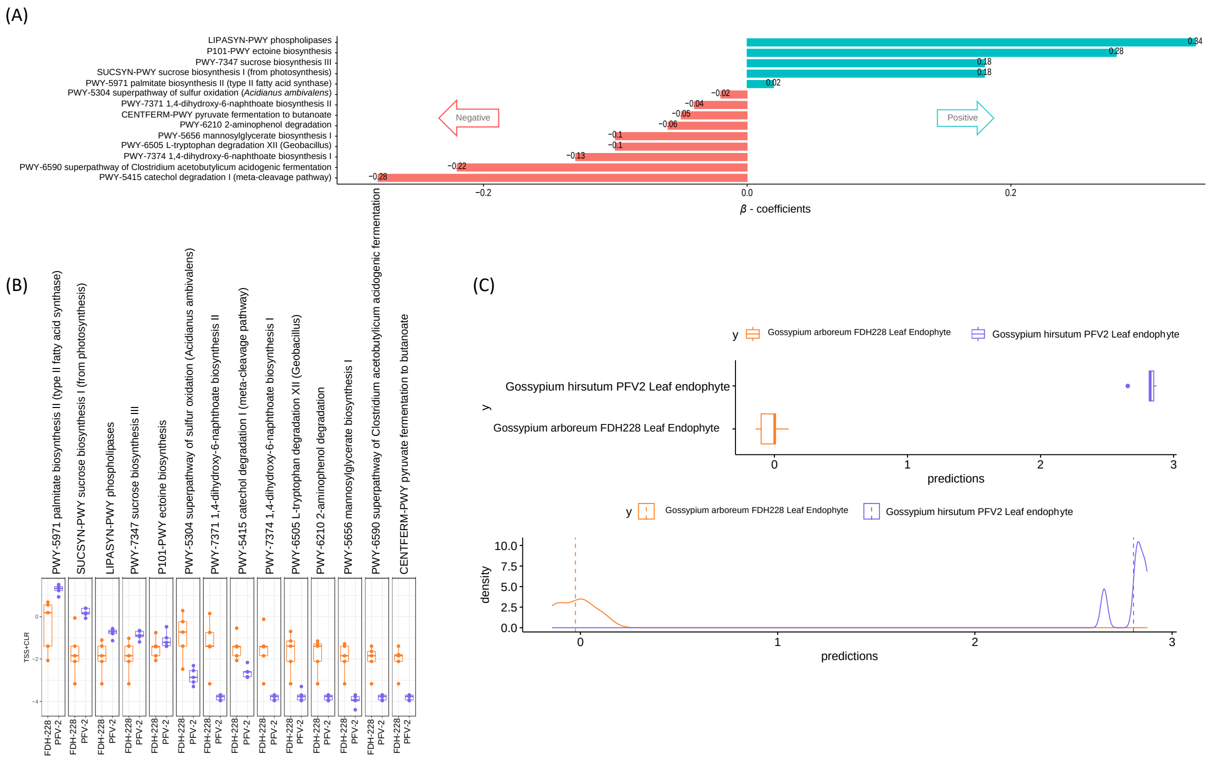

Supplementary Figure 14. CODA LASSO regression for *Gossypium hirsutum* PFV-2 and *Gossypium arboreum FDH-228* (Leaf Endophyte) for MetaCyc pathways A) β −coefficients returned from CODA-LASSO procedure as two disjoint sets (those that are positively related, and those that are negatively related with the temperature) B) The density plot returned from the CODA-LASSO segregates the two groups provides a graphical assessment of the classification accuracy (top: true; bottom: predicted from the procedure); C) Expression levels of microbes selected from the procedure.

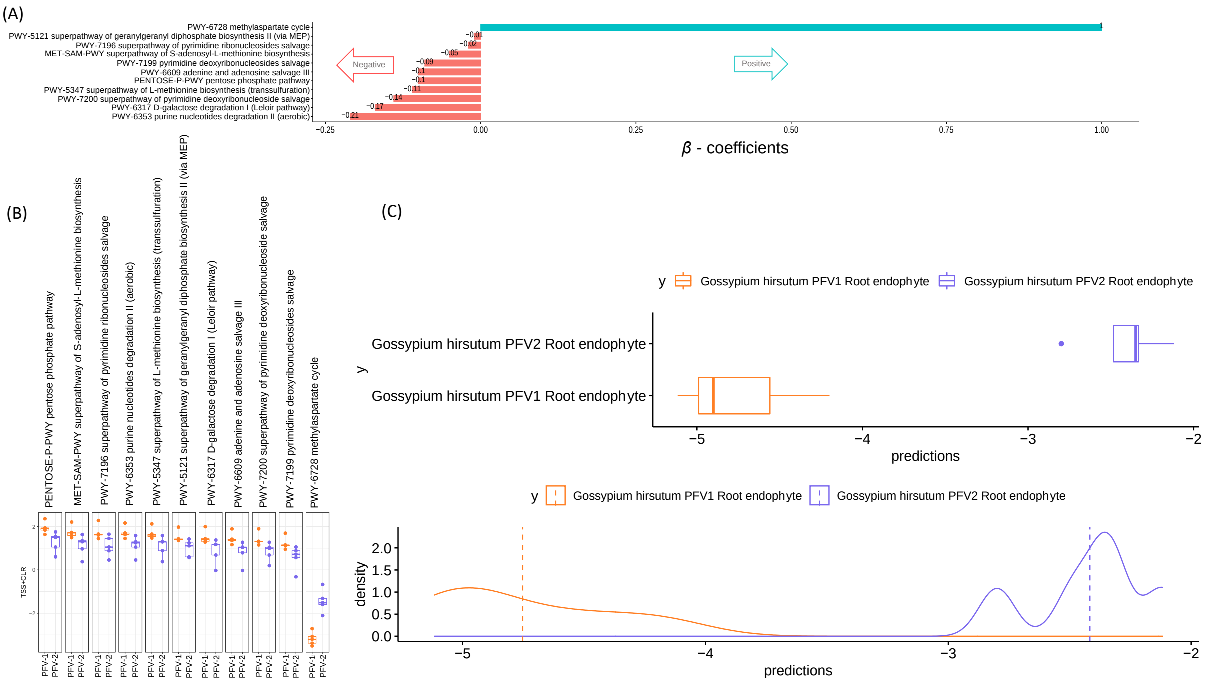

Supplementary Figure 15. CODA LASSO regression for *Gossypium hirsutum* PFV-1 and *Gossypium hirsutum* PFV-2 (Root Endophyte) for MetaCyc pathways A) β −coefficients returned from CODA-LASSO procedure as two disjoint sets (those that are positively related, and those that are negatively related with the temperature) B) The density plot returned from the CODA-LASSO segregates the two groups provides a graphical assessment of the classification accuracy (top: true; bottom: predicted from the procedure); C) Expression levels of microbes selected from the procedure.

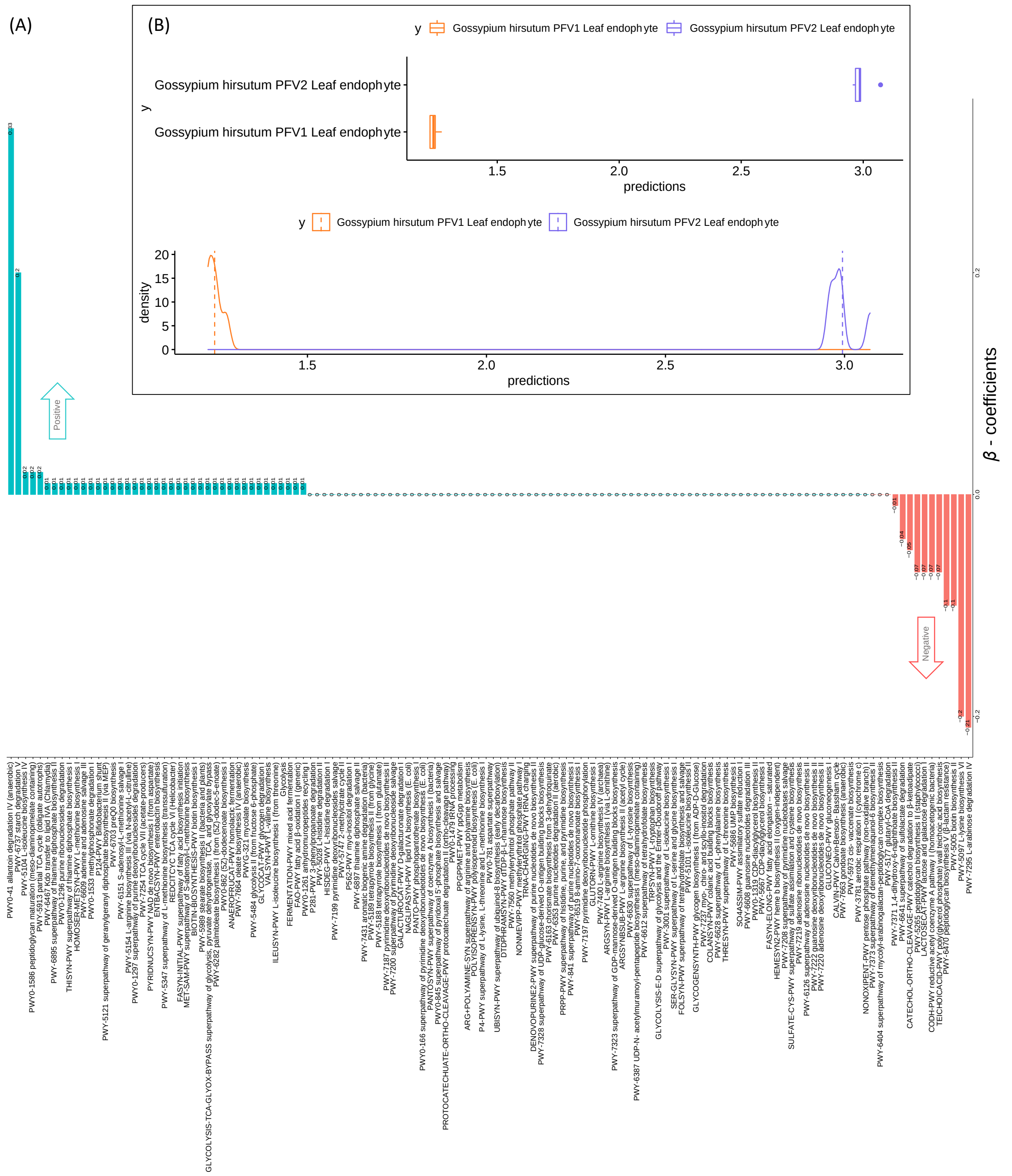

Supplementary Figure 16. CODA LASSO regression for *Gossypium hirsutum* PFV-1 and *Gossypium hirsutum* PFV-2 (Leaf Endophyte) for MetaCyc pathways A) β −coefficients returned from CODA-LASSO procedure as two disjoint sets (those that are positively related, and those that are negatively related with the temperature) B) The density plot returned from the CODA-LASSO segregates the two groups provides a graphical assessment of the classification accuracy (top: true; bottom: predicted from the procedure).

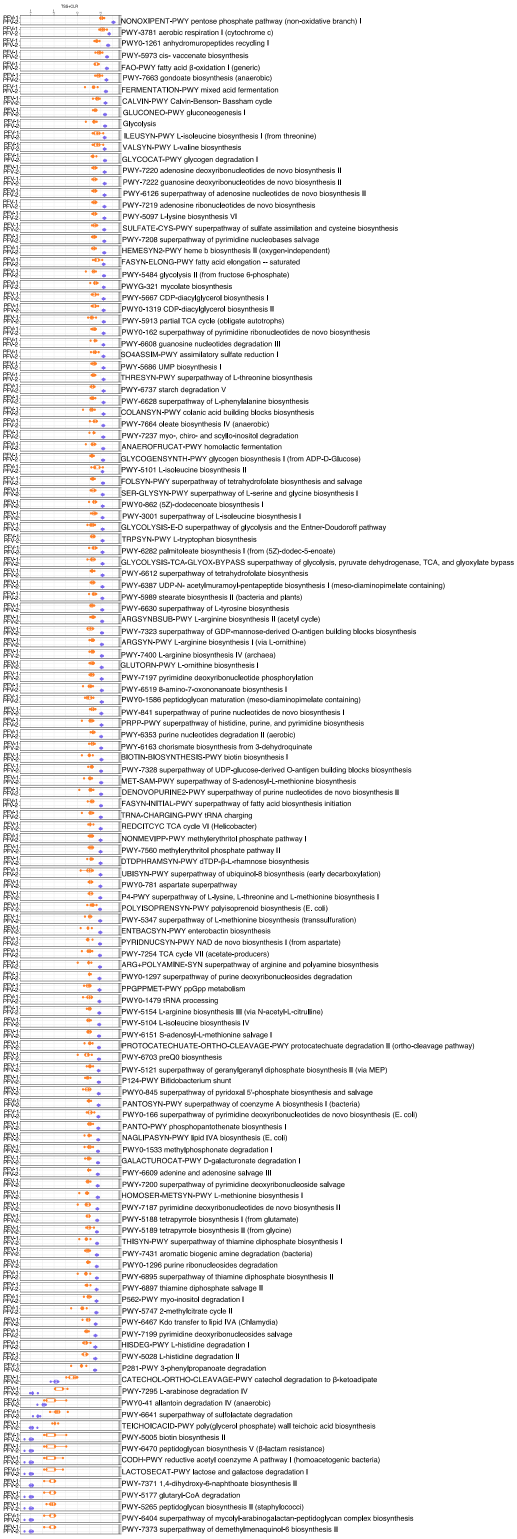

Supplementary Figure 17. CODA LASSO regression for *Gossypium hirsutum* PFV-1 and *Gossypium hirsutum* PFV-2 (Leaf Endophyte) for MetaCyc pathways. Expression levels of microbes selected from the procedure in Figure 2.5.5.

Supplementary Table 3: Top two most significantly positive and negative genera identified through CODA LASSO procedure in Supplementary Figures 5-11 that compared compartments for different cotton varieties. Their significance in previously published literature is also shown. The variety in which the expression is higher is annotated with up arrow.

Genus	Cotton Varieties Expression Trend	Compartment	Description
Methylophaga	PFV-1, FDH-228↑	Rhizosphere	In root microbiome of healthy seagrass [4] Isolated from rhizosphere of rice has PGPR activity [5]
Candidatus adlerbacteria	PFV-1, FDH-228 ↑	Rhizosphere	*C. Adlerbacteria* in rhizosphere of Arecanut palms (Yellow Leaf Disease) [6]
Gracilimonas	PFV-1, FDH-228 ↑	Rhizosphere	Dominant genus in halophyte [7] Rhizosphere of halophytes [8]
Chryseobacterium	PFV-1, FDH-228 ↑	Rhizosphere	Rhizosphere soil of Rhizoctonia solani Bare Patch Disease of Wheat [9]
Caulobacter	PFV-1, FDH-228 ↑	Leaf Epiphyte	Root microbiomes of *Arabidopsis* [10], *Citrullus* [11], Dioon [12] *Lavandula dentanta* L. [13], *Populus deltoides* [14], and *Z. mays* [15, 16]
Aeromonas	PFV-1, FDH-228 ↑	Leaf Epiphyte	Rhizosphere and endosphere of *Arabidopsis* [17]
Thermus	PFV-1, FDH-228 ↓	Leaf Epiphyte	Apple flower microbiome [18]
Gaiella	PFV-1, FDH-228 ↑	Leaf Epiphyte	Rhizosphere of resistant strawberry cultivars against soil-borne fungal pathogens [19]
Legionella	PFV-1, FDH-228 ↑	Leaf endophyte	Leaf microbiome of radish (*Raphanus sativus*), lettuce (*Lactuca sativa*), and pakchoi (*Brassica chinensis*) [20]
Deinococcus	PFV-1, FDH-228 ↑	Leaf endophyte	Native-grown *N. attenuata* plant roots [21]

			Tomato leaf microbiome [22]<br>Pear and apple bark [23]
Ahniella	FDH-228, PFV-2 ↑	Rhizosphere	Isolated from sandy soil near a stream [24]
Nocardia	FDH-228, PFV-2 ↑	Rhizosphere	Isolated from a root nodule of an Alnus glutinosa plant growing in Leazes Park, Newcastle upon<br>Tyne, UK [25]<br>Rhizosphere of desert plant *Calotropis procera* [26]
Gemmatimonas	PFV-2, FDH-228 ↑	Rhizosphere	Rhizosphere of mono-cropped system [27]<br>Rhizosphere microbiome of Jerusalem artichoke [28]<br>Anaerobic–aerobic sequential batch reactor [29]<br>Rhizosphere microbiome of Rhizoma Alismatis (Chinese medicinal herb) [30]<br>Rotation soil of chilli pepper-banana [31]<br>Surface water of a stream<br>in the Zackenberg Valley in High Arctic Greenland [32]<br>Freshwater Swan Lake in the western Gobi Desert [33]
BD2-11_terrestrial group	PFV-2, FDH-228 ↑	Rhizosphere	Hypersaline sodalakes [34]
Aureimonas	FDH-228, PFV-2 ↑	Leaf Epiphyte	Endorhizosphere of sunflower plants [35]<br>Rice seed microbiome [36, 37]
Pontibacter	PFV-2, FDH-228 ↑	Leaf Epiphyte	Saline-sandy area soil microbiome [38]

Rubellimicrobium	PFV-2, FDH-228 ↑	Leaf Epiphyte	Saline-sandy area soil microbiome [38]
Bradyrhizobium	PFV-2, FDH-228 ↑	Leaf endophyte	Soybean rhizosphere [39]<br>Canola rhizosphere [40]<br>Metarhizium treated root tissue of beans [41]
Johnsonella	FDH-228, PFV-2 ↑	Leaf endophyte	Not available for plant microbiome
Caulobacteraceae	PFV-2, FDH-228 ↑	Leaf endophyte	Rhizosphere microbiome of Solanaceae eggplant resistant varieties against bacterial wilt resistance [42]
Arsenophonus	PFV-1, PFV-2 ↑	Leaf endophyte	Pesticide treated tea leaf microbiome [43]<br>Insect pathogen [44]
Alishewanella	PFV-1, PFV-2 ↑	Leaf endophyte	Core pine nut microbiome [45]
Solibacillus	PFV-2, PFV-1 ↑	Leaf endophyte	Cowpea soil microbiome [46]<br>Healthy tomato rhizosphere soil [47]
Longimicrobiaceae	PFV-2, PFV-1 ↑	Leaf endophyte	Potato rhizosphere microbiome [48, 49]
Bdellovibrio	PFV-2, FDH-228 ↑	Rhizosphere	Citrus root-associated microbiome [50]<br>Rhizosphere of resistant variety [51]

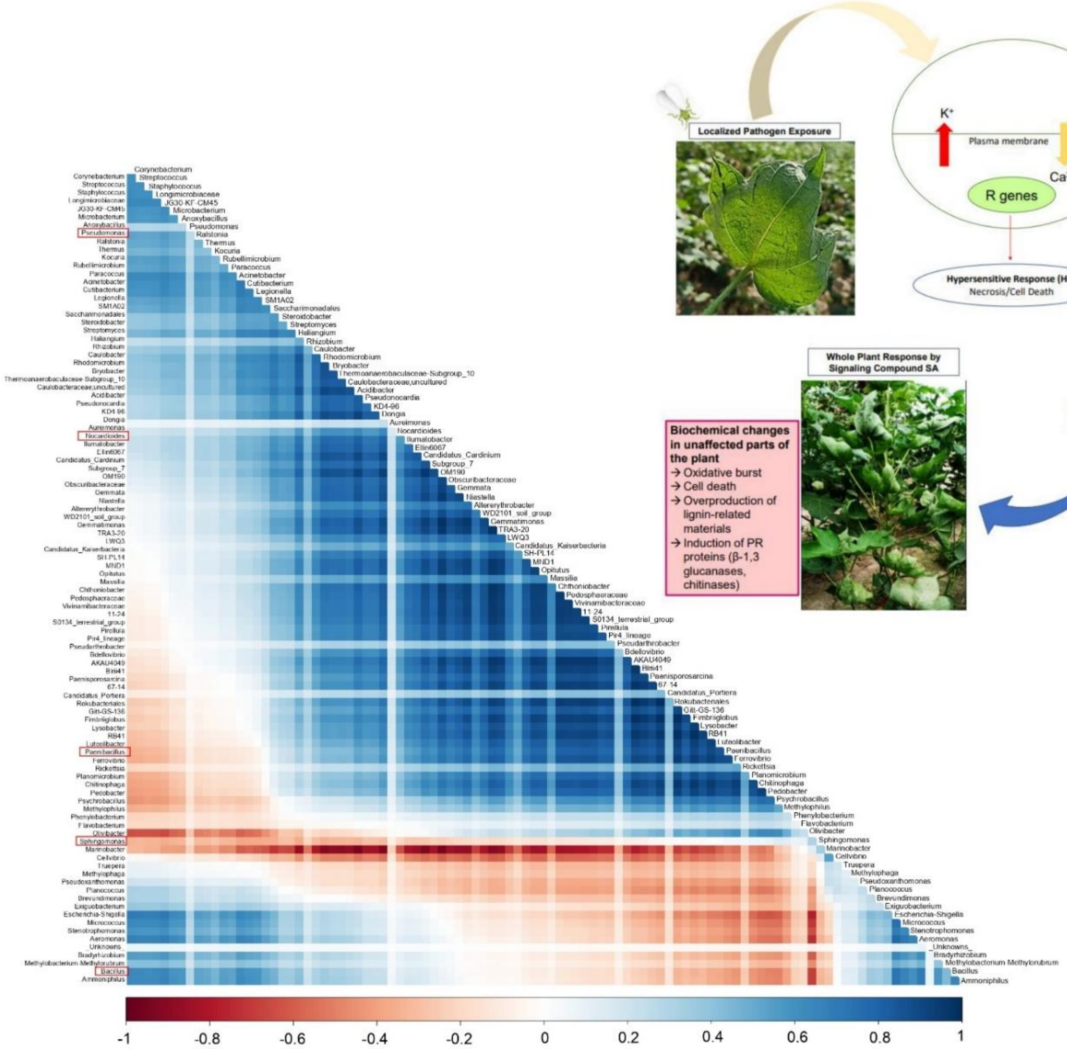

Supplementary Figure 18. Co-occurrence relationship between microbes recovered from the residual covariance matrix Σ that are not explained by the environmental covariates in the GLLVM model in Figure 4. Here, blue represent the positive correlation, and red represent the negative relationship. The highlighted bacteria are capable of SA production.

Supplementary Table 4: Bacterial Strains isolated from different plant compartments of CLCuD susceptible, partially tolerant, and resistant cotton varieties. This table shows their isolate codes, morphological characteristics, indole acetic acid and salicylic acid production results.

Plant Variety	Plant Compartment	Isolate Code	Shape	Colour	Elevation	Margin	Appearance	SA production	IAA production	16S rRNA based identification
PFV-2	Leaf epiphyte	V2 lep 10-8	Circular	white	flat	entire	opaque glossy	X	✓	-
		V2 lep 10-10	circular	dark pink	flat	entire	very small	X	X	-
		V2 lep 10-6	Circular	yellow	raised	entire	smooth, transparent	X	✓	-
	Leaf endophyte	V2 len 3-2	Circular	white	raised	entire	small, opaque	X	✓	-
		V2 len 3-3	Circular	neon lime	flat	entire	small, translucent	X	✓	-
		V2 len 10-6	Circular	yellow	flat	entire	transparent	X	✓	-
		V2 len 10-5	Circular	yellow	raised	entire	transparent	X	✓	-
		V2 len 10-2	Irregular	Dark yellow	flat	undulate	opaque	X	✓	-
		V2 len 6-1	Circular	white	flat	entire	small	X	✓	-
	Rhizosphere	V2 rhi 1-1	Circular	White	raised	Entire	Opaque glossy	X	✓	-
		V2 rhi 7-1	Irregular	Off white	flat	Undulate	opaque	X	✓	-
	Root endophyte	V2 ren 3-2	Filiform	Translucent white	flat	undulate	very small	X	X	-
PFV-1	Leaf epiphyte	EPI.PFV1.P1.TSA.C1	Circular	Yellow Inner	raised	entire	Small	X	✓	-
		EPI.PFV1.P3.TSA.C2	Irregular	Light yellow	convex	umbonate	Small	X	✓	-
		EPI.PFV1.P3.TSA.C1	Circular	Orange	convex	entire	Large	X	✓	-

	Leaf endophyte	ENDO.PFV1.P1.TSA.C1	Irregular	Pure white	flat	umbonate	Large	X	✓	-
		ENDO.PFV1.P3.TSA.C3	Circular	Off white outer	raised	entire	Small	X	✓	-
		ENDO.PFV1.P1.TSA.C2	Circular	Dull white	raised	entire	Small	X	✓	-
		ENDO.PFV1.P3.TSA.C2	Circular	Cream white	convex	entire	small	X	✓	-
		Rh.pfv1.P9.TSA.C2	Circular	White	Convex	Entire	Smooth	X	✓	-
		Rh.PFV1.P6.TSA.C1	Circular	Yellow	Flat	Entire	Viscid	X	✓	-
		RT En.PFV1.P3.TSA.C3	Irregular	Milky white	Convex	Undulate	Viscid	X	✓	-
		RT En PFV1.P3.TSA.C1	Irregular	Off white	Flat	Undulate	Smooth	X	✓	-
FDH-228	Leaf epiphyte	FHAP2.6	Circular	Pink	Convex	Entire	smooth,moist & coccibacilus	✓	✓	uncultured *Serratia spp.*
		FHAP7.7	Circular	Yellow	Convex	Entire	smooth,moist & rods	✓	✓	*Bacillus spp.*
		FHAP1.8	Circular	Off white	Raised	Entire	glistery,dry & rods	X	✓	-
	Leaf endophyte	FHAN2.7	Circular	Lemon	Convex	Entire	Glistery, moist & rod	X	✓	-
		FHAN2.8	Circular	Milky	Convex	Entire	Smooth, dry & rod	✓	X	*Fictibacillus spp.*
	Rhizosphere	Rh. P1. -5. C1	Circular	Yellow	Raised	Entire	Small	X	X	-
		Rh. P1. -5. C2	Circular	Pure White	Raised	Entire	Small	X	X	-

Supplementary Table 5: Soil Classification and Chemical Characteristics

**Soil Characteristic**	**Values**
**pH**	**7.43**
**EC (µScm^{-1})**	**99.52**
**Organic matter (%)**	**3.140**
**Total Kjeldhal Nitrogen (%)**	**0.028**
**Total Phosphorus P_2O_5 (%)**	**0.070**
**Total Potassium (%)**	**0.290**
**Total Calcium (%)**	**0.450**
**Total Magnesium (%)**	**0.380**
**Total Sodium (%)**	**8.710**
**Total Manganese (mg/Kg)**	**12.50**
**Soil texture**	**Sandy Loam**

Supplementary References:

[1] Herlemann, D. P., Labrenz, M., Jürgens, K., Bertilsson, S., Waniek, J. J., & Andersson, A. F. (2011). Transitions in bacterial communities along the 2000 km salinity gradient of the Baltic Sea. The ISME journal, 5(10), 1571-1579.

[2] Monga, D., Kumar, R., & Kumar, M. (2005). Detection of DNA-A and satellite (DNA-β) in cotton leaf curl virus (CLCuV) infected weeds and cotton plants using PCR technique. Journal of Cotton Research and Development, *19*(1), 105-108.

[3] Akhtar, K. P., Haidar, S., Khan, M. K. R., Ahmad, M., Sarwar, N., Murtaza, M. A., & Aslam, M. (2010). Evaluation of *Gossypium* species for resistance to cotton leaf curl Burewala virus. Annals of applied biology, 157(1), 135-147.

[4] Martin, B. C., Alarcon, M. S., Gleeson, D., Middleton, J. A., Fraser, M. W., Ryan, M. H., ... & Kilminster, K. (2020). Root microbiomes as indicators of seagrass health. FEMS Microbiology Ecology, 96(2), fiz201.

[5] Bal, H. B., & Adhya, T. K. (2021). Alleviation of submergence stress in rice seedlings by plant growth-promoting rhizobacteria with ACC deaminase activity. Frontiers in Sustainable Food Systems, 5, 606158.

[6] Paulraj, S., Bhat, R., Rajesh, M. K., Ramesh, S.V., Priya, U.K., Pandian, T. P. R., Hegde, V., and Chowdappa, P. 2021. Microbiome-mediated Rhizosphere Nitrogen Transformation Cycle (RNTC) potentially underlies the disease severity in Arecanut Yellow Leaf Disease (YLD): Insights from metagenomics. In: PLACROSYM XXIV (Eds.) Dhanapal, K., Kumar, K. P., Shadanaika, Ali, M.A.A., Varghese, J. J., Saju, K.A., Oommen, M., and Thiyagarajan, P., Indian Cardamom Research Institute/Indian Society for Plantation Crops, Kasaragod, Kerala. pp. 221-222.

[7] Gao, L., Huang, Y., Liu, Y., Mohamed, O. A. A., Fan, X., Wang, L., ... & Ma, J. (2022). Bacterial community structure and potential microbial coexistence mechanism associated with three halophytes adapting to the extremely hypersaline environment. Microorganisms, 10(6), 1124.

[8] Li, Y., Kong, Y., Teng, D., Zhang, X., He, X., Zhang, Y., & Lv, G. (2018). Rhizobacterial communities of five co-occurring desert halophytes. PeerJ, 6, e5508.

[9] Yin, C., Hulbert, S. H., Schroeder, K. L., Mavrodi, O., Mavrodi, D., Dhingra, A., ... & Paulitz, T. C. (2013). Role of bacterial communities in the natural suppression of Rhizoctonia solani bare patch disease of wheat (Triticum aestivum L.). Applied and Environmental Microbiology, 79(23), 7428-7438.

[10] Lundberg, D. S., Lebeis, S. L., Paredes, S. H., Yourstone, S., Gehring, J., Malfatti, S., ... & Dangl, J. L. (2012). Defining the core *Arabidopsis thaliana* root microbiome. Nature, 488(7409), 86-90.

[11] Ren, R., Yang, X., Xu, J., Zhang, M., Liu, G., & Yao, X. (2019). Genome-wide identification and analysis of GDSL-type esterases/lipases in watermelon (Citrullus lanatus).

[12] Gutiérrez-García, K., Bustos-Díaz, E. D., Corona-Gómez, J. A., Ramos-Aboites, H. E., Sélem-Mojica, N., Cruz-Morales, P., ... & Cibrián-Jaramillo, A. (2019). Cycad coralloid roots contain bacterial communities including cyanobacteria and Caulobacter spp. that encode niche-specific biosynthetic gene clusters. Genome Biology and Evolution, 11(1), 319-334.

[13] Pereira, S. I. A., Monteiro, C., Vega, A. L., & Castro, P. M. (2016). Endophytic culturable bacteria colonizing Lavandula dentata L. plants: isolation, characterization and evaluation of their plant growth-promoting activities. Ecological Engineering, 87, 91-97.

[14] Brown, S. D., Klingeman, D. M., Lu, T. Y. S., Johnson, C. M., Utturkar, S. M., Land, M. L., ... & Pelletier, D. A. (2012). Draft genome sequence of Rhizobium sp. strain PDO1-076, a bacterium isolated from Populus deltoides.

[15] Naveed, M., Mitter, B., Reichenauer, T. G., Wieczorek, K., & Sessitsch, A. (2014). Increased drought stress resilience of maize through endophytic colonization by Burkholderia phytofirmans PsJN and Enterobacter sp. FD17. Environmental and Experimental Botany, 97, 30-39.

[16] Gao, J., Luo, M., Peng, H., Chen, F., & Li, W. (2019). Characterization of cadmium-responsive MicroRNAs and their target genes in maize (*Zea mays)* roots. BMC Molecular Biology, 20(1), 1-9.

[17] He, D., Singh, S. K., Peng, L., Kaushal, R., Vílchez, J. I., Shao, C., ... & Zhang, H. (2022). Flavonoid-attracted Aeromonas sp. from the Arabidopsis root microbiome enhances plant dehydration resistance. The ISME Journal, 16(11), 2622-2632.

[18] Shade, A., McManus, P. S., & Handelsman, J. (2013). Unexpected diversity during community succession in the apple flower microbiome. MBio, 4(2), e00602-12.

[19] Lazcano, C., Boyd, E., Holmes, G., Hewavitharana, S., Pasulka, A., & Ivors, K. (2021). The rhizosphere microbiome plays a role in the resistance to soil-borne pathogens and nutrient uptake of strawberry cultivars under field conditions. Scientific Reports, 11(1), 1-17.

[20] Li, W. J., Li, H. Z., An, X. L., Lin, C. S., Li, L. J., & Zhu, Y. G. (2022). Effects of manure fertilization on human pathogens in endosphere of three vegetable plants. Environmental Pollution, 314, 120344.

[21] Santhanam, R., Oh, Y., Kumar, R., Weinhold, A., Luu, V. T., Groten, K., & Baldwin, I. T. (2017). Specificity of root microbiomes in native‐grown Nicotiana attenuata and plant responses to UVB increase Deinococcus colonization. Molecular Ecology, 26(9), 2543-2562.

[22] Toju, H., Okayasu, K., & Notaguchi, M. (2019). Leaf-associated microbiomes of grafted tomato plants. Scientific reports, 9(1), 1-11.

[23] Arrigoni, E., Antonielli, L., Pindo, M., Pertot, I., & Perazzolli, M. (2018). Tissue age and plant genotype affect the microbiota of apple and pear bark. Microbiological research, 211, 57-68.

[24] Hwang, W. M., Ko, Y., Kim, J. H., & Kang, K. (2018). Ahniella affigens gen. nov., sp. nov., a gammaproteobacterium isolated from sandy soil near a stream. International Journal of Systematic and Evolutionary Microbiology, 68(8), 2478-2484.

[25] Nouioui, I., Ghodhbane-Gtari, F., Pötter, G., Klenk, H. P., & Goodfellow, M. (2023). Novel species of Frankia, Frankia gtarii sp. nov. and Frankia tisai sp. nov., isolated from a root nodule of Alnus glutinosa. Systematic and Applied Microbiology, 46(1), 126377.

[26] Ramadan, A. M., Nazar, M. A., & Gadallah, N. O. (2021). Metagenomic analysis of rhizosphere bacteria in desert plant Calotropis procera. Geomicrobiology Journal, 38(5), 375-383.

[27] Li, P., Liu, J., Saleem, M., Li, G., Luan, L., Wu, M., & Li, Z. (2022). Reduced chemodiversity suppresses rhizosphere microbiome functioning in the mono-cropped agroecosystems. Microbiome, 10(1), 1-15.

[28] Yue, Y., Shao, T., Long, X., He, T., Gao, X., Zhou, Z., ... & Rengel, Z. (2020). Microbiome structure and function in rhizosphere of Jerusalem artichoke grown in saline land. Science of the Total Environment, 724, 138259.

[29] Zhang, H., Sekiguchi, Y., Hanada, S., Hugenholtz, P., Kim, H., Kamagata, Y., & Nakamura, K. (2003). Gemmatimonas aurantiaca gen. nov., sp. nov., a gram-negative, aerobic, polyphosphate-accumulating micro-organism, the first cultured representative of the new bacterial phylum Gemmatimonadetes phyl. nov. International journal of systematic and evolutionary microbiology, 53(4), 1155-1163.

[30] Wei, C., Gu, W., Tian, R., Xu, F., Han, Y., Ji, Y., ... & Wu, W. (2022). Comparative analysis of the structure and function of rhizosphere microbiome of the Chinese medicinal herb Alisma in different regions. Archives of Microbiology, 204(7), 448.

[31] Hong, S., Jv, H., Lu, M., Wang, B., Zhao, Y., & Ruan, Y. (2020). Significant decline in banana Fusarium wilt disease is associated with soil microbiome reconstruction under chilli pepper-banana rotation. European Journal of Soil Biology, 97, 103154.

[32] Zeng, Y., Nupur, Wu, N., Madsen, A. M., Chen, X., Gardiner, A. T., & Koblížek, M. (2021). Gemmatimonas groenlandica sp. nov. is an aerobic anoxygenic phototroph in the phylum Gemmatimonadetes. Frontiers in microbiology, 11, 606612.

[33] Zeng, Y., Feng, F., Medová, H., Dean, J., & Koblížek, M. (2014). Functional type 2 photosynthetic reaction centers found in the rare bacterial phylum Gemmatimonadetes. Proceedings of the National Academy of Sciences, 111(21), 7795-7800.

[34] Vavourakis, C. D., Andrei, A. S., Mehrshad, M., Ghai, R., Sorokin, D. Y., & Muyzer, G. (2018). A metagenomics roadmap to the uncultured genome diversity in hypersaline soda lake sediments. Microbiome, 6(1), 1-18.

[35] Nemr, R. A., Khalil, M., Sarhan, M. S., Abbas, M., Elsawey, H., Youssef, H. H., ... & Hegazi, N. A. (2020). “In situ similis” culturing of plant microbiota: a novel simulated environmental method based on plant leaf blades as nutritional pads. Frontiers in Microbiology, 11, 454.

[36] Eyre, A. W., Wang, M., Oh, Y., & Dean, R. A. (2019). Identification and characterization of the core rice seed microbiome. Phytobiomes Journal, 3(2), 148-157.

[37] Midha, S., Bansal, K., Sharma, S., Kumar, N., Patil, P. P., Chaudhry, V., & Patil, P. B. (2016). Genomic resource of rice seed associated bacteria. Frontiers in microbiology, 6, 1551.

[38] Jalal, R. S., Sheikh, H. I., Alotaibi, M. T., Shami, A. Y., Ashy, R. A., Baeshen, N. N., ... & Baeshen, M. N. (2022). The Microbiome of Suaeda monoica and Dipterygium glaucum From Southern Corniche (Saudi Arabia) Reveals Different Recruitment Patterns of Bacteria and Archaea. Frontiers in Marine Science, 9, 865834.

[39] Meena, R. S., Vijayakumar, V., Yadav, G. S., & Mitran, T. (2018). Response and interaction of Bradyrhizobium japonicum and arbuscular mycorrhizal fungi in the soybean rhizosphere. Plant Growth Regulation, 84, 207-223.

[40] Floc’h, J. B., Hamel, C., Laterrière, M., Tidemann, B., St-Arnaud, M., & Hijri, M. (2021). Inter-kingdom networks of Canola microbiome reveal Bradyrhizobium as keystone species and underline the importance of bulk soil in microbial studies to enhance Canola production. Microbial ecology, 1-16.

[41] Barelli, L., Waller, A. S., Behie, S. W., & Bidochka, M. J. (2020). Plant microbiome analysis after Metarhizium amendment reveals increases in abundance of plant growth-promoting organisms and maintenance of disease-suppressive soil. PLoS One, 15(4), e0231150.

[42] Jiang, G., Zhang, Y., Gan, G., Li, W., Wan, W., Jiang, Y., ... & Dini-Andreote, F. (2022). Exploring rhizo-microbiome transplants as a tool for protective plant-microbiome manipulation. ISME Communications, 2(1), 10.

[43] Cernava, T., Chen, X., Krug, L., Li, H., Yang, M., & Berg, G. (2019). The tea leaf microbiome shows specific responses to chemical pesticides and biocontrol applications. Science of the Total Environment, 667, 33-40.

[44] Darby, A. C., Choi, J. H., Wilkes, T., Hughes, M. A., Werren, J. H., Hurst, G. D. D., & Colbourne, J. K. (2010). Characteristics of the genome of Arsenophonus nasoniae, son‐killer bacterium of the wasp Nasonia. Insect Molecular Biology, 19, 75-89.

[45] Fay, M., Salazar, J. K., Ramachandran, P., & Stewart, D. (2021). Microbiomes of commercially-available pine nuts and sesame seeds. Plos one, 16(6), e0252605.

[46] de Sousa Lopes, L., Mendes, L. W., Antunes, J. E. L., de Souza Oliveira, L. M., Melo, V. M. M., de Araujo Pereira, A. P., ... & Araujo, A. S. F. (2021). Distinct bacterial community structure and composition along different cowpea producing ecoregions in Northeastern Brazil. Scientific Reports, 11(1), 1-12.

[47] Lee, S. M., Kong, H. G., Song, G. C., & Ryu, C. M. (2021). Disruption of Firmicutes and Actinobacteria abundance in tomato rhizosphere causes the incidence of bacterial wilt disease. The ISME journal, 15(1), 330-347.

[48] Hou, Q., Wang, W., Yang, Y., Hu, J., Bian, C., Jin, L., ... & Xiong, X. (2020). Rhizosphere microbial diversity and community dynamics during potato cultivation. European Journal of Soil Biology, 98, 103176.

[49] Pascual, J., García-López, M., Bills, G. F., & Genilloud, O. (2016). Longimicrobium terrae gen. nov., sp. nov., an oligotrophic bacterium of the under-represented phylum Gemmatimonadetes isolated through a system of miniaturized diffusion chambers. International journal of systematic and evolutionary microbiology, 66(5), 1976-1985.

[50] Zhang, Y., Xu, J., Riera, N., Jin, T., Li, J., & Wang, N. (2017). Huanglongbing impairs the rhizosphere-to-rhizoplane enrichment process of the citrus root-associated microbiome. Microbiome, 5, 1-17.

[51] Jiang, G., Zhang, Y., Gan, G., Li, W., Wan, W., Jiang, Y., ... & Dini-Andreote, F. (2022). Exploring rhizo-microbiome transplants as a tool for protective plant-microbiome manipulation. ISME Communications, 2(1), 10.
